# Comparative Genomics of a Bacterivorous Green Alga Reveals Evolutionary Causalities and Consequences of Phago-Mixotrophic Mode of Nutrition

**DOI:** 10.1093/gbe/evv144

**Published:** 2015-07-29

**Authors:** John A. Burns, Amber Paasch, Apurva Narechania, Eunsoo Kim

**Affiliations:** Sackler Institute for Comparative Genomics and Division of Invertebrate Zoology, American Museum of Natural History, New York, NY

**Keywords:** Chloroplastida, *Cymbomonas*, green algae, mixotrophy, phagocytosis

## Abstract

*Cymbomonas tetramitiformis*—a marine prasinophyte—is one of only a few green algae that still retain an ancestral particulate-feeding mechanism while harvesting energy through photosynthesis. The genome of the alga is estimated to be 850 Mb–1.2 Gb in size—the bulk of which is filled with repetitive sequences—and is annotated with 37,366 protein-coding gene models. A number of unusual metabolic pathways (for the Chloroplastida) are predicted for *C. tetramitiformis*, including pathways for Lipid-A and peptidoglycan metabolism. Comparative analyses of the predicted peptides of *C. tetramitiformis* to sets of other eukaryotes revealed that nonphagocytes are depleted in a number of genes, a proportion of which have known function in feeding. In addition, our analysis suggests that obligatory phagotrophy is associated with the loss of genes that function in biosynthesis of small molecules (e.g., amino acids). Further, *C. tetramitiformis* and at least one other phago-mixotrophic alga are thus unique, compared with obligatory heterotrophs and nonphagocytes, in that both feeding and small molecule synthesis-related genes are retained in their genomes. These results suggest that early, ancestral host eukaryotes that gave rise to phototrophs had the capacity to assimilate building block molecules from inorganic substances (i.e., prototrophy). The loss of biosynthesis genes, thus, may at least partially explain the apparent lack of instances of permanent incorporation of photosynthetic endosymbionts in later-divergent, auxotrophic eukaryotic lineages, such as metazoans and ciliates.

## Introduction

Chloroplastida (or Viridiplantae) is one of the major eukaryotic lineages, comprising green algae and land plants (Adl et al. 2012). The group is rich in diversity, including about 370,000 described species (Melkonian 1990; Plant List 2013), and is responsible for about half of the total global primary production (Kirchman 2012). Together, Chloroplastida, Rhodophyta, and Glaucophyta constitute Archaeplastida, the group characterized by having a plastid that is bound by two membranes, which is the basis for classifying them as primary plastids (i.e., direct descendants of endosymbiotic cyanobacteria) (Archibald 2009). The three archaeplastid lineages are assumed by many to have originated from a shared endosymbiotic event and to form a monophyletic group. However, data, especially those bearing on nucleocytoplasmic traits, thus far have not been able to unambiguously support the “Archaeplastida” hypothesis (Mackiewicz and Gagat 2014; Stiller 2014).

**Fig. 1.**
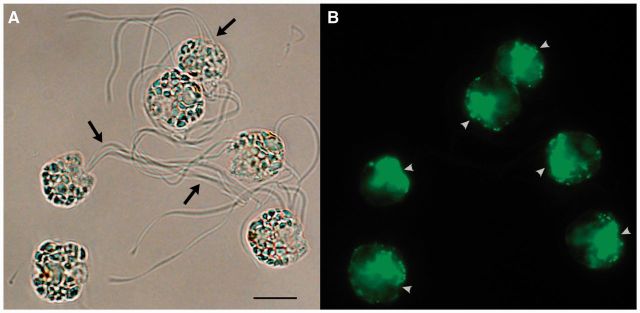
— *Cymbomonas tetramitiformis* cells stained with Alexa Fluor 488 phalloidin, which has a high affinity to F-actin. (*A*) (bright-field) Each cell bears four flagella (arrows). (*B*) (fluorescence, FITC channel) Phalloidin stain is concentrated at the cell’s anterior (arrowheads), where particle ingestion takes place. Scale bar = 10 µm.

Of the primary-plastid-containing eukaryotes, only a few are known to be phagocytotic, despite the presumption that phagocytotic engulfment of a photo-symbiont was necessary for the evolution of a photosynthetic organelle (Maruyama and Kim 2013). Most primary-plastid-bearing eukaryotes appear therefore to have lost the capacity to feed on bacteria or other large particulate matter, presumably as phototrophy took over the primary nutritive role after plastids were acquired (Raven et al. 2009; Cavalier-Smith 2013). No red algae or glaucophytes are known to be phagocytotic; however, some “early-diverging” green algae have been suggested to be phago-mixotrophic based on their internal cell morphology (Moestrup et al. 2003; Maruyama and Kim 2013). Of these, the marine tetraflagellate *Cymbomonas tetramitiformis* was definitively confirmed to engulf bacteria by transmission electron microscopy (Maruyama and Kim 2013). This green alga (fig. 1) appears to utilize a tubular channel to transport particles from the exterior environment into a permanent acidic vacuole, where digestion takes place (Maruyama and Kim 2013). Note that internalization of bacteria into root cells has been reported from some flowering plants (e.g., Leborgne-Castel et al. 2010; Paungfoo-Lonhienne et al. 2010); however, it is structurally different from green algal phagocytosis (e.g., absence/presence of a feeding channel) and thus likely represents a derived trait (also see Cavalier-Smith 2013), possibly stemming from inherent properties of the eukaryotic cell membrane.

From a broad evolutionary perspective, phagocytosis is restricted to the eukaryotes and is absent in prokaryotes (Cavalier-Smith 2002). The distribution of phagocytosis across a wide range of eukaryotic groups suggests that phagocytosis was present in the last eukaryotic common ancestor (LECA), although it has since been lost in several lineages after they diverged from LECA (Cavalier-Smith 2002; Yutin et al. 2009). The evolution of phagocytosis is considered a key element during eukaryogenesis (Cavalier-Smith 2002; Yutin et al. 2009), which may explain an observation that conserved proteins described for phagocytosis across diverse eukaryotic taxa thus far have heterologous functions (e.g., actin filaments and Rho family GTPases) and are not specific to phagocytosis (Chimini and Chavrier 2000; Yutin et al. 2009).

It should be emphasized that our current understanding of phagocytosis at the molecular and genomic levels is based on and limited to a small number of organisms, including the specialized phagocytotic cells of insects and mammals (e.g., macrophages), the amoebozoans *Dictyostelium discoideum* and *Entamoeba histolytica*, and the ciliate *Tetrahymena thermophila* (Okada et al. 2005, 2006; Gotthardt et al. 2006; Jacobs et al. 2006; Shevchuk et al. 2009; Boulais et al. 2010). In this study, we compared the *C. tetramitiformis* genome with those of phagocytotic and nonphagocytotic eukaryotes, to gain insight into both the alga itself and to the evolutionary genetics of nutritional modes.

## Materials and Methods

### Culturing and Phalloidin Staining

A culture of *C. tetramitiformis* sp. (PLY262) was obtained from the Plymouth Algal Culture Collection. The original strain was cocultured with a heterotrophic stramenopile of uncertain taxonomic identity; a clonal algal culture was established by single-cell isolation techniques. This reisolated culture strain was used for genome and transcriptome sequencing. The culture was maintained in f/2-Si medium (Guillard and Ryther 1962) at 16 °C under a 12-h light cycle with an average light intensity of 34 µmol·m^−^^2^·s^−^^1^.

Exponentially growing *C. tetramitiformis* cells were fixed by a mixture of formaldehyde and glutaraldehyde at final concentrations of 3.2% and 0.1%, respectively, for 10 min at room temperature (RT). The cells were permeabilized in 0.03% Triton X-100 and 100 µg/ml bovine serum albumin (BSA), buffered in phosphate buffered saline (PBS) for 5 min at RT. Alexa Fluor 488-phalloidin (Invitrogen) was then added to the cell solution according to the manufacturer’s recommendation. Phalloidin staining proceeded for 20 min at RT. The cells were resuspended in 50 mM glycine in PBS to quench autofluorescence from unreacted aldehydes. The cells were washed three times with PBS and the final pellet was resuspended in filter-sterilized seawater. In all relevant steps, cells were pelleted by centrifugation at 500 × g for 90 s. Microscopic imaging was done using a Zeiss Axiovert 100 inverted microscope (Carl Zeiss, Jena, Germany) equipped with an Olympus DP73 digital camera (Olympus, Tokyo, Japan).

### DNA and RNA Extraction and Sequencing

Total DNA of the alga was obtained from 50 ml of culture in a midexponential phase (∼200,000 cells·ml^−^^1^) using a PureLink Genomic DNA kit (Life Technologies; Carlsbad, CA). For RNA extraction, 50 ml of culture was mixed with an equal volume of RNAlater solution (Life Technologies) prior to being pelleted by centrifugation at 1,000 × g for 5 min. Total RNA was isolated using the TRIzol Plus RNA Purification System (Life Technologies). Quantitative and qualitative assessments of purified nucleic acids were conducted by Qubit assays (Life Technologies) and agarose gel electrophoresis, respectively.

The purified DNA and RNA materials were sent to GenomeQuebec and Beijing Genomics Institute, respectively, for library preparation and sequencing on the Illumina HiSeq 2000 platform. For genome sequencing, two libraries—one short insert library and one 3-kb mate-pair library—were prepared following the TruSeq preparation protocols. The transcriptome library for the alga was constructed using a TruSeq RNA preparation kit with polyA selection. These libraries were paired-end sequenced with a read length of up to 100 bp. Total numbers of 279,212,444 and 156,530,692 reads were generated for the short insert and mate-pair libraries, respectively. A total of 115,092,458 reads were obtained from the transcriptome library.

### Read Filtering and Genome Assembly

Read filtering was implemented prior to final assembly in an effort to avoid hybrid contigs (Phillippy et al. 2008; Claros et al. 2012). Reads corresponding to mitochondrial, chloroplast, or cocultured bacterial genomes were removed using the following five steps: 1) First, an initial genome assembly was done using the entire data set of short insert (fragment) and mate-pair libraries using AllPaths-LG (Gnerre et al. 2011). From the initial assembly, the two largest contigs were identified as partial or complete bacterial genomes by open reading frame (ORF) modeling and BLAST (Basic Local Alignment Search Tool) searches against the nr and nt databases on the NCBI (National Center for Biotechnology Information) webserver using default parameters. 2) Draft organellar genomes for the mitochondrion and chloroplast were assembled using the MITObim wrapper script (Hahn et al. 2013) (V 1.6) with the MIRA assembler (MIRA V 3.4.1.1) (Chevreux et al. 2004). The *Ostreococcus tauri* mitochondrion (Palenik et al. 2007) and *Pyramimonas parkae* chloroplast (Turmel et al. 2009) genomes were used as reference scaffolds. 3) The bacterial and organellar genomes were used to build a custom Kraken (Wood and Salzberg 2014) (V 0.10.2-beta) database containing all 31mers found in the bacterial and organellar assemblies (using Jellyfish [Marçais and Kingsford 2011] V 1.1.11). 4) The Kraken database from step 3 was used to filter the Illumina reads with the condition that at least ten 31mers from the Kraken database had to have an exact hit within a read to classify it as bacterial or organellar. Reads that were not classified as bacterial or organellar were retained for reassembly. 5) 3.1% of the total reads (4,381,732/139,606,222) were identified as organellar or bacterial in either or both of the forward and reverse reads and removed prior to reassembly using AllPaths-LG (supplementary fig. S1, Supplementary Material online). All computations were carried out on our in-house system with four Intel Xeon E7-4850 2-GHz processors and 1 TB RAM. This Whole Genome Shotgun project has been deposited at DDBJ/EMBL/GenBank under the accession LGRX00000000. The version described in this paper is version LGRX01000000

### Flow Cytometry Analysis of C. tetramitiformis Nuclei

DNA content analysis of *C. tetramitiformis* nuclei was performed at the Benaroya Research Institute at Virginia Mason by Dr K. Arumuganathan using a modified procedure from Arumuganathan and Earle (1991). Briefly, 1 ml of *C. tetramitiformis* culture was pelleted and resuspended by vortexing vigorously in 0.5 ml of a nuclear extraction solution (10 mM MgSO_4_.7H_2_O, 50 mM KCl, 5 mM HEPES, pH 8.0, 3 mM dithiothreitol, 0.1 mg·ml^−^^1^ propidium iodide, 1.5 mg·ml^−^^1^ DNAse free RNAse [Roche, Indionapolis, IN], 0.25% Triton X-100). The suspended solution that contained nuclei was filtered through 30-µm nylon mesh, and incubated at 37 °C for 30 min (for staining and RNA digestion) before flow cytometric analysis alongside with standard nuclei. The mean nuclear DNA content, measured in picograms, was based on 1,000 scanned nuclei.

### Genome Annotation—MAKER

The MAKER (Cantarel et al. 2008) (V 2.28) pipeline was used for structural annotation of the *C. tetramitiformis* genome. Gene model inputs to MAKER proceeded from an annotation pipeline that was adapted from a previous study (Kumar 2013). Evidence-based annotation of the genome was derived from a separate de novo assembled transcriptome for *C. tetramitiformis.* The transcriptome was assembled in Trinity (Grabherr et al. 2011) (V Trinity-r2012-10-05). MAKER was run iteratively, three times as follows. The first MAKER run added evidence-based annotation using the transcriptome assembly. The output from the first run was used to train the ab initio gene prediction algorithm SNAP (Korf 2004) (V 2006-07-28) in two iterations of the SNAP training algorithm. The second run of MAKER used the SNAP HMM for ab initio gene predictions. The output from the second MAKER run was used to train AUGUSTUS (Stanke and Waack 2003) (V 2.7). MAKER was then run a third time incorporating the AUGUSTUS HMM for *C. tetramitiformis* to update the gene models.

### Functional Genome Annotation

Functional genome annotation was carried out by 1) A DELTA-BLAST (Boratyn et al. 2012) homology search, with an *e*-value cutoff of 1e^−^^5^, using the peptide sequence output from MAKER as query sequences and the curated UniProt-SProt peptides as the database. This first step assigned potential homologs to 60.6% (22,632 of 37,366) of the *C. tetramitiformis* peptides. 2) Because statistically significant similarity does not necessarily mean equivalent function (Rost 1999, 2002; Sander and Schneider 1991), for each BLAST hit, a “homology-derived structure of proteins” (HSSP) distance was calculated using the “DIST” relation (Rost 2002) of equation 3 in Rost (2002). DELTA-BLAST hits with a DIST score greater than 5 were retained. The dual annotation criteria resulted in putative functional annotation of 11,209 *C. tetramitiformis* peptides. UniProt-SProt (Gasteiger et al. 2001) fasta files and the DELTA-BLAST conserved domain database were downloaded on August 5, 2014. Custom Perl scripts were used to extract enzyme commission (EC) number annotations for *C. tetramitiformis* peptides from the uniprot gene association file gene_association.goa_uniprot (Ashburner et al. 2000) that was downloaded on August 7, 2014 based on the best hits to the UniProt-Sprot database that satisfied the HSSP scoring parameters.

### Conserved Eukaryotic Genes Mapping Approach

As a way to estimate how complete the genome is, Conserved Eukaryotic Genes Mapping Approach (CEGMA) (V 2.4) (Parra et al. 2007, 2009) was run on the *C. tetramitiformis* genome assembly as well as select genomes that were downloaded from publicly available sources (supplementary table S1, Supplementary Material online). The CEGMA analysis used geneid (V 1.4) (Blanco et al. 2007), genewise (V wise2.2.3-rc7) (Birney et al. 2004), HMMER (V 3.0) (Eddy 2009), and TBLASTN (V 2.2.25). CEGMA was run using default parameters with an option to allow for longer intron lengths. Intron and exon number and length statistics were obtained from the CEGMA output files using a custom Perl script. In addition to the default criteria, the *C. tetramitiformis* assembly was evaluated using the criteria previously used for the haptophyte *Emiliania huxleyi* genome (Read et al. 2013): Briefly, the search is based on BLASTP analysis with an *e*-value cutoff of 10^−^^5^ and a requirement of at least 50% alignment coverage against the CEGMA 468 core protein set.

### Metabolic Analysis—MetaCyc

MetaCyc (Caspi et al. 2008) is a freely available database containing 2,260 metabolic pathways of primary and secondary metabolisms in 2,600 organisms across all domains of life as of this writing. The weighted pathway scoring scheme from Kastenmuller et al. (2009), “score(p),” was adapted for use with the MetaCyc pathways in a custom Perl script. Incomplete EC annotations were removed from the *C. tetramitiformis* annotation and from the MetaCyc pathways such that only EC annotations with all four classification levels enumerated were retained for analysis (e.g., EC-3.1.4.13 would be retained whereas EC-3.1.4.- would be removed from the analysis). If an enzyme was present in *C. tetramitiformis* based on the functional genome annotation criteria discussed in “Functional Genome Annotation” section, it was considered present in the corresponding MetaCyc pathways. Presence/absence of pathways was determined by comparing pathway scores in *C. tetramitiformis* with those of three other green algae, *Chlamydomonas reinhardtii*, *O. tauri*, and *Micromonas pusilla.* False positives in the presence/absence list were eliminated by manually investigating each peptide in the respective organisms by BLASTP analysis.

The evolutionary histories of conspicuous metabolic peptides were investigated using phylogenetic analysis. Amino acid sequences were searched using DELTABLAST against the nr database (as well as others) with an *e*-value cutoff of 10^−^^5^. Up to the top 1,000 DELTABLAST hits were retained for subsequent phylogenetic analysis. Sequences were aligned in MAFFT (V 7.205) (Katoh and Standley 2013). Alignments were trimmed using trimAl (V 1.2rev59) (Capella-Gutiérrez et al. 2009). Maximum-likelihood (ML) analysis was performed using RAxML (V 8.1.11) (Stamatakis 2014) on the CIPRES XSEDE Portal (Miller et al. 2010) using the LG + I + G model, which was selected with ProTest (V 3.4) (Darriba et al. 2011). Bootstrapping was based on 1,000 replicates; every fifth bootstrap tree was used as a starting tree for ML analyses.

### Read Mapping and Repeat Identification

The algorithm Bowtie2 (Langmead and Salzberg 2012) (V 2.2.3) was used to map the reads from the fragment library back to the assembled genome using both forward and reverse reads and default parameters. The Bowtie output in “.sam” format was converted to a sorted .bam file using samtools (Li et al. 2009) (V 0.1.19-44428cd) and summary statistics were computed with QualiMap (García-Alcalde et al. 2012) (V 1.0).

RepeatMasker (Smit et al. 1996–2010) (V 4.0.5) was used to identify conserved repeats using the 20140131 version of the Repbase (Jurka et al. 2005) (GIRI) repeats library and the species flag option of “eukaryotes.” De novo repeats were identified using the RepeatModeler (V 1.0.8) (Smit and Hubley 2008–2010) package (RECON V 1.08 [Smit and Hubley 2008–2010], RepeatScout V 1.0.5 [Price et al. 2005], TRF V 4.07b [Benson 1999], and RMBlast V 2.2.28 [Smit and Hubley 2008–2010]). The RepeatModeler library was then used to find de novo repeats in the genome assembly.

### Genome Wide-Comparative Analysis

#### Selection of Taxa

For whole-genome comparisons, the BLASTP (V 2.2.28+) (Camacho et al. 2009) algorithm was used to query every predicted peptide in the *C. tetramitiformis* genome against the full complement of proteins in a group of organisms known to be phagocytotic: The amoebozoan *D. discoideum*, the parasitic amoebozoan *E. histolytica*, the fruit fly *Drosophila melanogaster*, the mouse *Mus musculus*, the apusomonad *Thecamonas trahens*, the ciliate *T. thermophila*, and the phago-phototrophic chlorarachniophyte *Bigelowiella natans*; and a group of organisms where an active form of phagocytosis has not been observed: The plants *Arabidopsis thaliana* (but see Leborgne-Castel et al. 2010; Paungfoo-Lonhienne et al. 2010) and *Oryza sativa*, the green alga *Ch. reinhardtii*, the red alga *Cyanidioschyzon merolae*, the yeast (fungus) *Saccharomyces cerevisiae*, and the chytrid fungus *Batrachochytrium dendrobatidis* (see supplementary table S1, Supplementary Material online, for the detailed version information of files used in this analysis). Proteins from each genome were considered “present” in the *C. tetramitiformis* genome if BLASTP hits to a *C. tetramitiformis* peptide matched the following two criteria: 1) An *e* value of 1e^−^^4^ or less (a more permissive *e* value than used for functional annotation due to the smaller—single genome size—database [Jones and Swindells 2002]) and 2) a DIST score > 5.

A list of proteins observed in purified phagosomes for *Mu. musculus*, *Dr. melanogaster*, and *D. discoideum* was obtained from Boulais et al. (2010), for *E. histolytica* from Okada et al. (2006), and for *T. thermophila* from Jacobs et al. (2006). A set of 1,591 unique phagosome proteins was obtained by combining unique phagosome components from each organism (shared phagosome proteins were discovered by BLAST of phagosome proteins to each genome).

#### Optimization of Inference Criteria for Phagocytosis-Related Proteins

Proteins associated with phagocytosis in *C. tetramitiformis* were inferred in the following procedures. First, sets of proteins shared between *C. tetramitiformis* and a phagocyte or *C. tetramitiformis* and a nonphagocyte were obtained by parsing BLAST results with custom scripts in the R programming environment (R-Core-Team 2014). If a given *C. tetramitiformis* peptide had a match in a given organism that exceeded the dual *e* value and HSSP criteria discussed above, it received a score of 1 (present) for that organism; if there was no match that met those criteria, it received a score of 0 (absent) for that organism (supplementary file S1, Supplementary Material online).

Next, organisms were grouped by their phagocytotic capacity and a group-wise presence score within the group of phagocytotic organisms and the group that does not complete phagocytosis was computed for each *C. tetramitiformis* peptide by considering the sum of presence scores within each group of organisms for each *C. tetramitiformis* peptide. For example, a presence score of 7 within the group of phagocytotic organisms meant that a gene that corresponds to that *C. tetramitiformis* peptide could be found within all seven phagocytotic organisms. Conversely, the same gene might have a presence score of 1 within the group of nonphagocytotic organisms meaning that only one of six nonphagocyte genomes carries the gene.

We reasoned that sets of peptides enriched in phagocytes would have a higher presence score among phagocytes than among nonphagocytes. The most stringent comparison between the two groups thus was *C. tetramitiformis* peptides with a score of 7 in phagocytes and a score of 0 in nonphagocytes. This most strict criteria, however, produced only a single peptide, likely due to the heterologous nature of genes that are involved in phagocytosis (e.g., Chimini and Chavrier 2000). Therefore, our approach was to identify sets of “enriched” genes in phagocytes when compared with nonphagocytes. The criteria for enrichment could be relaxed in two ways, by either relaxing the presence criteria in phagocytes (e.g., present in six or more phagocytes instead of present in all seven) or relaxing the absence criteria in nonphagocytes (e.g., absent from any five or more instead of absent from all six). All possible combinations of inclusion criteria from the two groups were considered for this analysis to identify an optimal search parameter for the study.

A custom scoring scheme was developed to find the optimal grouping strategy for revealing enriched genes among phagocytes or nonphagocytes. Briefly, the scoring scheme considered 1) the number of genes in the enrichment set, 2) the proportion of those genes found within the known phagosome, and 3) the distance between the phagosome proportion scores among phagocytes and nonphagocytes for a given grouping strategy.

The scoring scheme was derived from several observations. First, for each group (where a group was defined by presence criteria [i.e., present in seven of seven phagocytes, or six of seven, etc.]), the proportion of peptides with homology to known phagosome components versus the stringency of the comparison (absence criteria) (supplementary fig. S2, Supplementary Material online) showed that as the presence criteria was relaxed, the proportion of peptides with homology to known phagosome components was reduced, on average. Second, the number of peptides in the enrichment set versus stringency of the comparison (supplementary fig. S3, Supplementary Material online) showed that the average number of peptides in each set increased as the presence criteria was relaxed. Third, 4,305 *C. tetramitiformis* peptides share homology with known phagosome components. This number was used to weight sets of peptides such that a set of peptides derived from an optimal grouping strategy should include at least 4,305 peptides. Having too many or too few peptides in a set would incur a penalty. A weighted proportion score (wps) was thus calculated that included a penalty for distance from 4,305 peptides:

$$\text{wps}=\frac{\text{\#peptides }\;\text{of }\;\text{set}\;\text{in}\;\text{known}\;\text{phagosome}}{\text{\#peptides}\;\text{in}\;\text{set}}*100-\text{penalty}*\left(1-\frac{\text{\#peptides}\;\text{of}\;\text{set}\;\text{in}\;\text{known}\;\text{phagosome}}{4305}\right).$$

The penalty was calculated based on the relation:

$$\text{penalty}=[{\left(100^{\frac{1}{\left|{\text{log}}_{4305}\left(\frac{\text{min}}{4305}\right)\right|}}\right)}^{\left|{\text{log}}_{4305}\left(\frac{\text{\#peptides}}{4305}\right)\right|}]-1,$$

where the further from 4,305 the greater the penalty (supplementary fig. S3*B*, Supplementary Material online) with a minimum set defined by “min,” set as 30 for this analysis and #peptides as the number of peptides in the set in question. The penalty was also constrained such that it was set to equal 100 where the penalty plot exceeded 100 so that the penalty score could not exceed the maximum possible proportion score of 100. Weighted scores were plotted for each set (supplementary fig. S4*A*, Supplementary Material online). Finally, signal to noise was considered as true enrichment of phagosome components versus inclusion of unrelated peptides due to unknown correlations among groups of organisms. The signal was considered as a function of the distance between the weighted score for phagocytes and the weighted score for nonphagocytes. The distance between weighted scores for phagocytes and nonphagocytes was calculated (as in supplementary fig. S4*B*, Supplementary Material online) and used to compute a distance score (ds):

$$\text{ds}=\;{\text{wps}}_{\text{phagocytes}}*\frac{\left({\text{wps}}_{\text{phagocytes}}-{\text{wps}}_{\text{nonphagocytes}}\right)}{100}.$$

Distance scores were plotted for each possible grouping of phagocytes and a set of conditions emerged that optimized the number of peptides in a set, the proportion of those peptides found in the known phagosome, and the signal to noise distance between peptides enriched in phagocytes and those enriched under the same comparison parameters in nonphagocytes. For these analyses, the optimal grouping strategy was identified as *C. tetramitiformis* peptides that are found in six or seven of seven phagocyte genomes but are absent from three or more nonphagocyte genomes (supplementary fig. S5, Supplementary Material online, red arrowhead).

#### Gene Ontology Analyses

The set of 399 *C. tetramitiformis* peptides enriched among phagocytes and the set of 702 *C. tetramitiformis* peptides enriched among nonphagocytes were analyzed for gene ontology (GO) (Ashburner et al. 2000) term enrichment using the R package topGO (Alexa and Rahnenfuhrer 2010). The reference set for topGO enrichment analysis was the annotated set of 11,209 *C. tetramitiformis* peptides as described in “Functional Genome Annotation” section. Significant GO term enrichment was calculated using variations of the Fisher’s exact test built into the topGO package. Redundancy was eliminated from the resultant list of significant GO terms by excluding GO terms consisting of identical peptide lists in favor of the GO term with the lowest Fisher’s elim *P* value (Alexa and Rahnenfuhrer 2010). Organism-specific heat maps were generated in R (R-Core-Team 2014) using the heatmap.2 function in the “gplots” package (Warnes et al. 2013). Heat maps used the average presence score for all peptides associated with a given GO term within a given organism. GO terms were ranked by taking the average of the organism-specific presence scores across phagocytes or nonphagocytes for each GO term.

## Results

### General Assembly Statistics

Analysis of isolated nuclei of *C. tetramitiformis* by flow cytometry estimated the nuclear DNA content (that of the tetraflagellate *C. tetramitiformis* cell, which may be diploid) at 1.73 Gb ± 10.8 Mb—in other words, a genome size of 850 Mb. On the other hand, K-mer spectrum analysis of the sequencing reads of the alga suggested a genome size of 1.2 Gb. The unique (nonrepetitive) portion of the genome was estimated to be 270 Mb, by calculating the area under the curve around the peak K-mer coverage. The algal nuclear genome was assembled using the AllPaths-LG algorithm (Gnerre et al. 2011) to 40,241 scaffolds (assembled from 74,370 contigs greater than 1 kb in length) with a total assembled length of 264.5 Mb and an N50 scaffold size of 10 kb (table 1).

**Table 1:** Assembly Statistics for *Cymbomonas tetramitiformis.* Minimum Contig Size for Reporting Was 1,000 Bases

Statistic	Value
Number of contigs	74,370
Number of scaffolds	40,241
Total contig length	258,572,850
Total scaffold length, with gaps	264,540,525
N50 contig size (kb)	4.9
N50 scaffold size (kb)	10
% GC	52.53

### Repeat Structure of the Genome

Following assembly, the modal per base coverage of sequencing reads across the genome was found to be 19 × whereas the modal average coverage per scaffold was 21 × (supplementary fig. S6, Supplementary Material online). There were four scaffolds, however, with greater than 10,000 × average coverage, 23 scaffolds with greater than 5,000 × coverage, 435 scaffolds with > 1,000 × coverage, and 6,446 scaffolds with > 100 × coverage. Three of the scaffolds with > 10,000 × coverage contain putative protein-coding regions with significant homology to a chromodomain/chromobox peptide, an RNaseH domain, and a cytosine DNA methyltransferase; these are known to be associated with repetitive DNA elements, such as retrotransposons in other eukaryotic genomes (Kubis et al. 1998; Pavlopoulou and Kossida 2007; Gao et al. 2008; Schulman 2013). Of the 23 scaffolds with > 5,000 × coverage, 19 contain sequences that can be found in the transcriptome assembly. Although these scaffolds are overrepresented in the genome, the transcripts with related sequences do not show evidence of overexpression as a whole. Out of 50 transcripts with significant homology to overrepresented scaffolds (>1,000×), the median number of fragments per kilobase of transcript per million mapped reads (FPKM) value was 1.32 (with a range from 0.12 to 391.27). This is close to the overall median FPKM value of 1.42 for the whole transcriptome, suggesting tight regulation of genes in the repetitive regions.

Within the assembled genome (not accounting for sequencing depth), RepeatModeler (Smit and Hubley 2008–2010) identified 9,044 long terminal repeat (LTR) retrotranspoable elements totaling 5.2 Mb and 1.88% of the assembled genome (table 2). Additionally, 1,230 short interspersed nuclear elements (SINEs) and 8,261 long interspersed elements (LINEs) were identified, accounting for 0.1% and 1.31% of the assembled genome. RepeatModeler also identified a proportion of the assembled genome (18.04%) comprising interspersed repeats that could not be classified based on homology to known elements (table 2).

**Table 2:** Summary of Repeats Classified in the *Cymbomonas tetramitiformis* Genome Assembly

Element	Number of Elements^a^	Length Occupied (bp)	% of Sequence
SINEs	1,230	273,877	0.10
LINEs	8,261	3,685,274	1.31
LTR elements	9,044	5,283,877	1.88
DNA elements	10,795	2,492,999	0.89
Unclassified	224,860	50,747,125	18.04
Small RNA	987	216,436	0.08
Satellites	610	121,373	0.04
Simple repeats	50,617	2,867,598	1.02
Low complexity	3,855	203,268	0.07

^a^Most repeats fragmented by insertions or deletions have been counted as one element

### Genome Completeness

The CEGMA (Parra et al. 2007, 2009) algorithm was used as a measure of completeness of the *C. tetramitiformis* genome assembly, in comparison to genomes of select eukaryotes (table 3). Despite the large discrepancy in estimated genome size to assembly length, CEGMA was able to annotate 76.61% or 91.7% of the core eukaryotic genes in *C. tetramitiformis* using, respectively, either the default criteria or a separate set of criteria that was used for analysis of the haptophyte *Em. huxleyi* genome (Read et al. 2013). Other statistics associated with the CEGMA genes indicate that the *C. tetramitiformis* genome contains a comparable number of introns per gene and a comparable average intron length to the green alga *Ch. reinhardtii* (table 3).

**Table 3:** Summary Statistics of Genomes of *Cymbomonas tetramitiformis* and Other Select Eukaryotic Organisms

Higher Taxonomic Rank	Species	Genome Size (Mb)	Mean Number of Introns	Mean Intron Length	Mean CDS Length (bp)^a^	Predicted Peptide #
Chloroplastida	***Cymbomonas tet.*** ** PLY262**	[650–850]	**7.40**	**296.90**	**1,127.73**	**37,366**
Chloroplastida	*Arabidopsis thaliana*	135	7.65	157.97	1,269.48	27,416
Chloroplastida	*Chlamydomonas reinhardtii*	120	8.11	312.57	1,317.89	15,598
Chloroplastida	*Micromonas pusilla* C3	21.96	1.62	288.99	1,389.36	10,137
Chloroplastida	*Ostreococcus tauri*	12.56	0.87	258.55	1,284.81	7,603
Chloroplastida	*Physcomitrella patens*	480	6.65	247.27	1,255.47	32,273
Amoebozoa	*Dictyostelium discoideum*	34	1.62	143.50	1,293.31	12,257
Apusomonads	*Thecamonas trahens*	26.68	1.04	442.67	1,335.15	10,544
Cryptista	*Guillardia theta*	87.16	5.88	130.93	1,271.98	24,840
Haptophyta	*Emiliania huxleyi*	167.7	3.82	224.24	1,357.97	39,126
Opisthokonta	*Homo sapiens*	3,300	8.72	1,741.40	1,228.94	20,300
Opisthokonta	*Monosiga brevicollis*	41.6	7.17	148.44	1,257.32	9,196
Rhizaria	*Bigellowiella natans*	94.7	6.86	183.89	1,266.17	21,708
Rhodophyta	*Cyanidioschyzon merolae*	16.5	0.22	786.72	1,363.23	5,771
Stramenopiles	*Thalassiosira pseudonana*	32	1.26	257.52	1,385.35	11,242

Note.—Bold accents *Cymbomonas tetramitiformis* strain PLY262.

^a^Calculations are based on genes predicted by the CEGMA pipeline.

### Metabolic Pathway Analysis

Annotation of *C. tetramitiformis* peptides against the curated UniProt-SProt database resulted in functional annotation of 11,209 algal peptides (supplementary file S2, Supplementary Material online). For metabolic pathway analysis, the annotation pipeline produced 1,280 unique EC annotations representing 3,748 *C. tetramitiformis* proteins. Analyses of the EC annotations against MetaCyc metabolic pathways (Caspi et al. 2008) showed that the *C. tetramitiformis* assembly has complete or nearly complete components of key eukaryotic metabolic pathways (table 4 and supplementary file S3, Supplementary Material online).

**Table 4:** Key Pathways in *Cymbomonas tetramitiformis* Metabolic Analysis in Comparison to Three Reference Green Algae

Pathway	No. Enzymes in Pathway	No. Enzymes in *Cymbomonas tetramitiformis*	*Cymbomonas tetramitiformis* Score	*Chlamydomonas reinhardtii* Score	*Ostreococcus tauri* Score	*Micromonas pusilla* Score
Key pathways						
Calvin–Benson–Bassham cycle	11	11	1.00	1.00	1.00	1.00
Fatty acid & beta-oxidation I	6	6	1.00	1.00	1.00	1.00
Gluconeogenesis I	14	13	0.96	0.88	0.68	0.68
Glycogen biosynthesis I (from ADP-d-Glucose)	6	5	0.97	0.97	0.97	0.97
Glycolysis III (from glucose)	10	10	1.00	1.00	0.66	1.00
Pentose phosphate pathway	8	7	0.80	0.80	0.80	0.80
Superpathway of fatty acid biosynthesis II (plant)	12	10	0.87	0.83	0.83	0.83
TCA cycle I (prokaryotic)	11	9	0.92	0.94	1.00	0.94
Urea cycle	5	3	0.32	0.32	0.32	0.51
Pathways present in *Cymbomonas tetramitiformis* but absent from other algae						
2-Methylbutyrate biosynthesis	5	3	0.41	0.04	0.03	0.03
2-Oxobutanoate degradation I	3	2	0.70	0.00	0.00	0.00
3-Hydroxypropanoate 4-hydroxybutanoate cycle	15	8	0.44	0.04	0.03	0.03
Choline-o-sulfate degradation	3	3	1.00	0.15	0.15	0.15
CMP-KDO biosynthesis I	4	3	0.75	0.00	0.25	0.25
Ethylene biosynthesis I (plants)	3	3	1.00	0.21	0.21	0.03
Glutamate degradation IV	4	3	0.94	0.11	0.06	0.06
Glycogen degradation III	6	4	0.86	0.15	0.15	0.15
Isoleucine degradation I	5	4	0.55	0.10	0.09	0.09
Lysine degradation II (mammalian)	4	4	1.00	0.16	0.00	0.00
Nitrate reduction V (assimilatory)	4	3	0.48	0.06	0.06	0.06
Propionyl CoA degradation	3	2	0.70	0.00	0.00	0.00
Sucrose degradation IV (sucrose phosphorylase)	6	4	0.91	0.14	0.14	0.14
Superpathway of (KDO)2-lipid A biosynthesis	12	8	0.67	0.17	0.08	0.08
Superpathway of lipopolysaccharide biosynthesis	15	8	0.49	0.12	0.06	0.06
Terminal o-glycans residues modification	7	4	0.57	0.00	0.00	0.00
Succinate fermentation to butyrate	6	3	0.42	0.00	0.00	0.00
d-Galactarate degradation I	4	2	0.56	0.00	0.00	0.00
Pathways present in other algae but absent from *Cymbomonas tetramitiformis*						
Ethanol degradation IV	3	1	0.06	1.00	0.24	0.24
Mannitol cycle	5	1	0.01	0.05	0.05	0.65
Mevalonate pathway II (archaea)	6	2	0.03	0.91	0.86	0.86
Pyruvate fermentation to ethanol I	3	1	0.23	1.00	0.87	0.87
Superpathway of acetate utilization and formation	3	1	0.12	1.00	0.76	0.76

Note.—The score for each organism refers to the weighted pathway score.

When pathway scores for *C. tetramitiformis* were compared with scores computed for three other green algae (i.e., *Ch. reinhardtii*, *O. tauri*, and *M. pusilla*), it was predicted that 19 pathways that are present in *C. tetramitiformis* are absent from the three other green algae (table 4). Conversely, the analysis suggests that *C. tetramitiformis* lacks five metabolic pathways that are present in one or more of the other green algae (table 4). The metabolic pathways that *C. tetramitiformis* maintains to the exclusion of other green algae include a lipid A synthesis pathway and portions of a predominantly archaeal carbon fixation pathway (i.e., the 3-hydroxypropanoate/4-hydroxybutanoate cycle) (table 4). *Cymbomonas tetramitiformis* is predicted to contain a complete set of proteins necessary for peptidoglycan synthesis, which is uncommon in the Chloroplastida (Cayrou et al. 2012).

### Phagocytosis Peptides

**Fig. 2.**
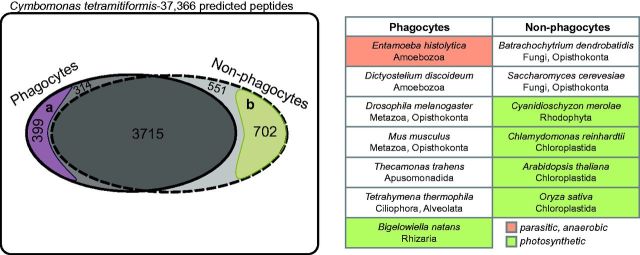
— The genome content analysis of *Cymbomonas tetramitiformis* in comparison to phagocyotic and nonphagocyotic eukaryotes as listed in the inset table. About 10% of *C. tetramitiformis* peptides (3,715/37,366) are present at least in 11 of all 13 organisms considered in the analysis. A total of 399 *C. tetramitiformis* peptides were identified to be enriched among phagocytes. Conversely, 702 *C. tetramitiformis* peptides were enriched among nonphagocytes; as discussed in the text, this set, more precisely, represents peptides that tend to get lost during obligatory phago-heterotrophic mode of nutrition.

Peptides related to phagocytosis in *C. tetramitiformis* were inferred through a comparative genomics approach. When the entire complement of predicted *C. tetramitiformis* peptides was compared with those of other phagocytes and nonphagocytes, 399 *C. tetramitiformis* peptides emerged that were present in *C. tetramitiformis* and at least six (out of seven total) other phagocyte genomes, but absent in three or more nonphagocyte genomes (fig. 2 and supplementary file S4, Supplementary Material online). In the control analysis (the reverse of what is described above: Present in *C. tetramitiformis* and at least five of six total nonphagocyte genomes, but absent from three or more other phagocyte genomes), 702 *C. tetramitiformis* peptides that are enriched among nonphagocyte sets were identified (fig. 2 and supplementary file S5, Supplementary Material online). The proportion of the phagocyte-enriched set of peptides that share homology with known phagosome components is 51% (or 205/399), which is greater than the value for the control set (17%). Our search criteria (i.e., peptides present in all [*N*] or *N* − 1 phagocytes and absent in at least three nonphagocytes) were identified by a weighted scoring scheme that gauges both the sensitivity and inclusiveness of peptides in the set as described in Materials and Methods.

**Fig. 3.**
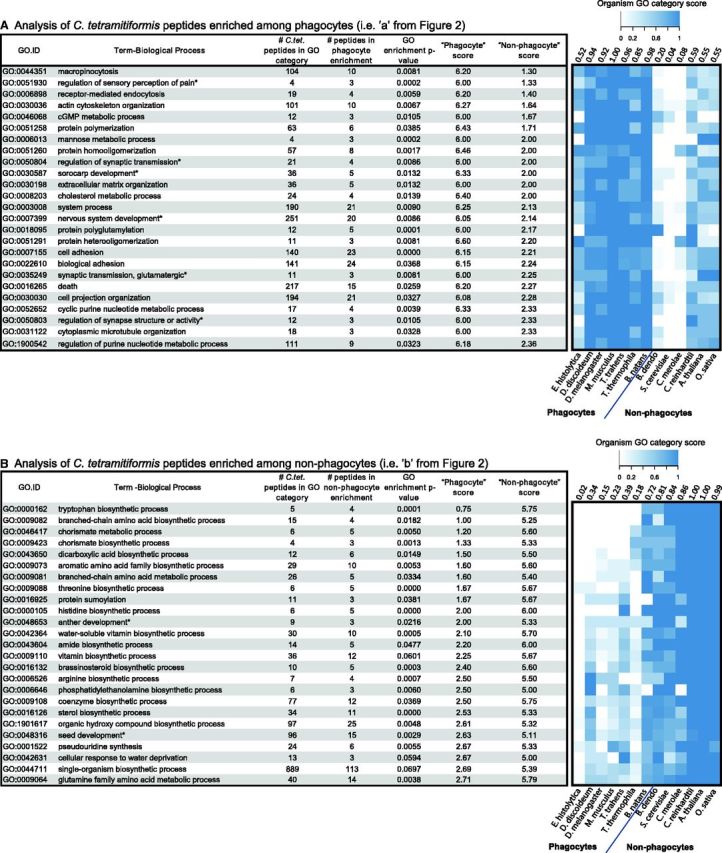
— GO term enrichment analysis of two sets of *Cymbomonas tetramitiformis* peptides identified from comparative analyses (fig. 2). (*A*) The top 25 Biological Process GO terms from *C. tetramitiformis* peptides enriched among phagocytes. The GO terms were sorted by presence in nonphagocyte genomes from low to high. (*B*) The top 25 Biological Process GO terms from *C. tetramitiformis* peptides enriched among nonphagocytes. The GO terms were sorted by presence in phagocyte genomes from low to high. The heat map to the right of each table represents the presence of all the peptides in that GO category in each organism. The number of above each column of the heat map is the average presence score for all 25 GO terms in that organism. Asterisks (*) indicate GO terms associated with organism-specific processes. These are due to the multiorganism annotation.

An interesting pattern emerged when the sets of *C. tetramitiformis* peptides enriched among phagocytes or nonphagocytes were analyzed by function. The 399 *C. tetramitiformis* peptides associated with phagocyte genomes revealed, as predicted, significant enrichment of several functional categories strongly associated with feeding by phagocytosis. These include macropinocytosis, receptor-mediated endocytosis, actin cytoskeleton organization, cGMP metabolic process, cell adhesion, and cell projection organization (fig. 3 and supplementary file S6, Supplementary Material online). In contrast, for the 702 *C. tetramitiformis* peptides that are enriched among nonphagocytes (i.e., fungi and nonphagotrophic eukaryotic phototrophs), functional enrichment was, predictably, not strongly associated with phagosome components, but was rather enriched in metabolic and in particular biosynthetic processes. The top enriched biological processes included biosynthesis of amino acids (e.g., tryptophan, threonine, and histidine) and intermediate compounds (e.g., chorismate for amino acid synthesis) (fig. 3 and supplementary file S7, Supplementary Material online).

## Discussion

### Characteristics of the *C. tetramitiformis* Genome

The genome size as estimated both by physical analysis of *C. tetramitiformis* nuclei and by estimation based on sequencing reads is between 850 Mb and 1.2 Gb. This is among the largest green algal genomes sequenced to date (Merchant et al. 2007; Palenik et al. 2007; Worden et al. 2009), and is over twice the median genome size (368.5 MB) of examined green algae of diverse taxonomic lineages (Kapraun 2007). *Cymbomonas tetramitiformis* has a genome that is about 100 times the size of the marine prasinophytes *M. pusilla* and *O. tauri* (table 3); it is, however, smaller than some other green algae, such as the freshwater streptophyte alga *Euastrum pectinatum*, which has an estimated genome size of 23 Gb (Kapraun 2007). Based on k-mer spectrum analysis, the total repeat content of the *C. tetramitiformis* genome is estimated to be about 77% of the genome. This value is greater than the repeat content of the model green alga *Ch. reinhardtii* (20%) (Prochnik et al. 2010), and much greater than the prasinophyte green algae *O. tauri* and *M. pusilla* (<10% repeats) (Palenik et al. 2007; Worden et al. 2009), but on the order of recent estimates of repeat content in other larger genomes, such as the human genome (66–69%) (de Koning et al. 2011) and the genome of the parasitic parabasalid *Trichomonas vaginalis* (>65%) (Carlton et al. 2007). Note that the total repeat content of the assembled genome of *C. tetramitiformis* is calculated to be 23%; due to the fragmented assembly and overrepresented scaffolds, much of the repetitive content of the genome likely remains unassembled and/or collapsed into fewer scaffolds as suggested by the presence of scaffolds with extreme coverage. The large estimated proportion of unassembled genome observed for *C. tetramitiformis* is similar to the draft assembly of the dinoflagellate *Symbiodinium minutum*; a gene rich 616 Mb was assembled out of an estimated genome size of 1.5 Gb despite having its genome sequenced at 37-fold coverage (Shoguchi et al. 2013). Although the exact mechanism for maintaining such a large genome is unknown for *C. tetramitiformis*, we did find evidence of at least one expressed retrotransposon in the transcriptome data (FPKM 6.05), which may be suggestive of active rearrangement and expansion of the mixotrophic green algal genome (Neumann et al. 2003).

The *C. tetramitiformis* assembly was annotated with 37,366 protein models, 18,698 (50%) of which are grouped into 8,354 ortholog groups by orthoMCL analysis (Chen et al. 2006; Fischer et al. 2011). This is similar to the *S. minutum* genome, which has 41,740 protein models and 8,031 orthologous groups (Shoguchi et al. 2013). Compared with *C. tetramitiformis*, genomes of other green algae tend to have fewer predicted peptides (7,725 for *O. tauri*; 10,137 for *M. pusilla*; 15,598 for *Ch. reinhardtii*); however, *Ch. reinhardtii* has a comparable number of orthologous groups (8,361) to that of *C. tetramitiformis* (Chen et al. 2006). Completeness metrics such as CEGMA and metabolic pathway analysis indicated that the *C. tetramitiformis* draft assembly has captured a significant portion of the protein-coding regions of the algal genome.

### Noteworthy Pathways of C. tetramitiformis Metabolism

#### Peptidoglycan Biosynthesis

Peptidoglycan (PG) is a major cell-wall component of bacteria, including cyanobacteria from which plastids evolved (Raven 2003). Of plastid-bearing eukaryotes, glaucophytes are the only group that have been shown to have a PG cell wall between the inner and outer membranes of their plastids (Raven 2003). Corroborating this, the genome of the glaucophyte *Cyanophora paradoxa* carries a number of genes related to PG synthesis (Bhattacharya et al. 2014). Rather unexpectedly, MetaCyc metabolic pathway analysis suggests that the *C. tetramitiformis* genome encodes putative homologs for all enzymes required for PG synthesis (van Heijenoort 2001). Furthermore, *C. tetramitiformis* encodes a set of three proteins—the glycosyltransferases GT28 (Pon1) and GT51 (MurG) and a glucoside hydrolase family protein (GH103 in this case)—that were suggested to be key indicators of the presence of PG metabolism according to Cayrou et al. (2012). At least two others within the Chloroplastida, including the prasinophyte green alga *M. pusilla* and the bryophyte *Physcomitrella patens*, encode the same set of three PG-related proteins. Phylogenetic analyses of individual protein data sets (supplementary figs. S7–S9, Supplementary Material online) suggest that the predicted PG metabolism of the Chloroplastida is evolutionarily conserved and originated from their common ancestor. Interestingly, the chloroplastidan PG metabolism only partially overlaps with that of the glaucophyte *Cy. paradoxa*; unlike green algae and plants, which have GH103 (supplementary fig. S7, Supplementary Material online), *Cy. paradoxa* has instead GH23, and, furthermore, its GT28 protein is not closely related to the chloroplastidan proteins (supplementary fig. S8, Supplementary Material online). Thus, of the three key components of PG metabolism, only GT51 proteins of the two lineages are closely related to each other, which together are sister to a clade comprising cyanobacteria (supplementary fig. S9, Supplementary Material online). Localization and other biochemical investigations are needed to determine whether PG is indeed synthesized and/or localized to the plastid membrane in *C. tetramitiformis* and other members of the Chloroplastida.

#### Lipid A and 2-Keto-3-Deoxymanno-Octulosonic Acid Biosynthesis

Lipid A is best known for its role in the outer membrane of Gram-negative bacteria, both as an anchor for lipopolysaccharides (Erridge et al. 2002) and as a potent antigen of mammalian immune systems (Pulendran et al. 2001). Lipid A is typically glycosylated with 2-keto-3-deoxymanno-octulosonic acid (KDO) sugars, such that both biosynthesis pathways would tend to be found in concert (Trent et al. 2006). The genome of *C. tetramitiformis* contains KDO and lipid A synthesis pathways similar to those described in the plant *A. thaliana*, in which the protein activities of the pathways were seen largely to localize to the mitochondria (Li et al. 2011; Smyth and Marchant 2013). In contrast, according to our analyses, in *Ch. reinhardtii*, *O. tauri* and *M. pusilla*, these pathways appear to be absent (table 4). These results are consistent with previous studies, in which the KDO sugars were shown to be the major components of the scales of prasinophyte green algae such as *Mesostigma viride* and *Scherfferlia dubia* (Becker et al. 1991) and a component of land plant cell walls (Bar-Peled et al. 2012), but were absent from naked or nonscaly green algae, including *M. pusilla* and *Ch. reinhardtii* (Becker et al. 1991). Together, these data suggest that the KDO and lipid A synthesis pathways were present in the last common ancestor of Chloroplastida, which was predictably scaly, but have been lost on multiple occasions.

#### Some Other Oddities

*Cymbomonas tetramitiformis* is distinct from other members of the Chloroplastida in that it has a putative glucoamylase, which can completely degrade starch and glycogen to β-d-glucose monomers (Sauer et al. 2000). This enzyme does not appear to be encoded in the genome of any other member of the Chloroplastida. Phylogenetic analysis shows that the *C. tetramitiformis* glucoamylase is closely related to those found in members of other eukaryotic groups, such as the apusomonad *Th. trahens*, the choanoflagellates *Monosiga brevicolis* and *Salpingoeca rosetta*, the cryptomonad *Goniomonas avonlea*, and the chlorarachniophyte *B. natans*, which together are related to the glucoamylase of the pathogenic bacterium *Legionella* (Herrmann et al. 2011) (supplementary fig. S10, Supplementary Material online). Diverse fungal taxa and one red alga, *Galdieria sulphuraria*, also encode a glucoamylase; these, however, have separate evolutionary origins from other eukaryotic glucoamylases (supplementary fig. S11, Supplementary Material online). Consistent with phylogeny, a clade comprising *C. tetramitiformis*, choanoflagellates, an apusomonad, and a cryptomonad is characterized by the presence of a trefoil-like domain—which may imply “extracellular” function (Braun et al. 2009)—at the N-terminus of glucoamylase; this domain, however, is not present in proteins of *B. natans*, fungi, and bacteria. Given its close relatedness to homologs found among phagotrophs (and absence of its homolog in nonphagotrophic green algal and plant relatives), the *C. tetramitiformis* glucoamylase may be involved in degradation of foods in the algal phagosome.

Another oddity of *C. tetramitiformis* lies in the presence of a putative homolog of the enzyme 4-hydroxybutyryl-CoA dehydratase, a key enzyme of the 3-hydroxypropanoate/4-hydroxybutanoate cycle for autotrophic carbon fixation in archaea and aminobutyrate fermentation in bacteria (Berg et al. 2010; Könneke et al. 2014). The presence of this enzyme has only been noted in one other eukaryotic organism, the photosynthetic stramenopile *Aureococcus anophagefferens* (Könneke et al. 2014); these two eukaryotic sequences are closely related to each other in phylogeny (supplementary fig. S12, Supplementary Material online)*.* Although the *C. tetramitiformis* enzyme does not appear to function in carbon fixation, as other genes necessary to complete the cycle have been not found, it may be used in other aspects of carbohydrate metabolism, as has been suggested previously (Berg et al. 2007).

### A Comparative Genomics Approach to the Inference of Phagocytosis-Related Genes in a Green Alga

Inference of phagocytosis-associated genes in *C. tetramitiformis* was based on whole genome-to-genome comparisons of the alga to phagocytotic and nonphagocytotic eukaryotic organisms (fig. 2). Our approach used the predicted peptides of the phago-mixotrophic green alga *C. tetramitiformis* as a baseline, and found subsets of *C. tetramitiformis* peptides with homologs enriched in phagocytic organisms compared with nonphagocytic organisms. The approach was a genome-wide version of more specific attempts to identify core phagosome components (Yutin et al. 2009; Boulais et al. 2010). Because phagocytosis-associated proteins are likely not limited to feeding function only, as has been previously suggested (Carter et al. 2004; Boulais et al. 2010; Deschamps et al. 2013), our approach was to look for peptides that tend to be retained in phagocytes, but can be lost in nonphagocytes. Based on peptide search parameters as described in Materials and Methods, we identified and analyzed a set of the *C. tetramitiformis* peptides that are present in almost all (six or seven out of seven) phagocytes but are absent in three or more nonphagocytes.

From this approach, a total of 399 *C. tetramitiformis* peptides were found to be enriched among phagocytes when compared with nonphagocytes. GO (Ashburner et al. 2000) enrichment analysis of these peptides revealed several categories containing peptides that have known functional overlap in phagocytosis such as the biological processes macropinocytosis, cell adhesion, cell projection organization, receptor-mediated endocytosis, and actin cytoskeleton organization (Maniak 2002) as well as other categories containing phagocytosis-related peptides despite seemingly unrelated category names. For example, the category, “regulation of sensory perception of pain,” contains sodium channel protein type 11 subunit alpha (Scn11a) which was shown to have a role in regulating phagocytosis and acidification of late endosomes in human macrophages (Carrithers et al. 2007). Summarizing GO term enrichment analyses, the general pattern emerged that the genomes of organisms that can complete phagocytosis are enriched in cell surface molecules, in particular cell adhesion and cell junction molecules, and proteins that regulate actin remodeling and vesicle trafficking through cell signaling or direct interactions with the cytoskeleton (fig. 3 and supplementary figs. S12–S14, Supplementary Material online).

It should be noted that some of the known phagosome components, such as actin, did not show up in our analysis due to their involvement in diverse cellular processes (e.g., cytokinesis in case of actin; Deschamps et al. 2013) Further, as our approach was geared toward shared components in phagocytosis across a range of eukaryotes, lineage-specific phagocytosis peptides predictably could not be inferred. Phagosome proteomics and other molecular studies for *C. tetramitiformis* will be necessary to identify lineage-specific components in phagocytosis as well as to confirm the inferences made in this study.

### Retention of Obligatory Phagotrophy as a Driving Force behind Loss of Biosynthesis Pathway Genes

On the other side of the analysis, which was initially intended as a control experiment, a number of biosynthetic pathways were found to be enriched among nonphagocytes as well as phago-mixotrophic algae (fig. 3). In particular, a number of genes related to amino acid biosynthesis pathways were found to be depleted among obligatory phagotrophs and metazoans. For instance, all of the top ten GO entries for the peptide set from the control experiment are related to amino acid synthesis, including synthesis of trypophan, threonine, and histidine as well as intermediate compounds, such as chorismate (fig. 3). The results are consistent with previous studies that showed that amino acid auxotrophy is related to feeding of other organisms or adaptation to parasitic life style (Payne and Loomis 2006). Our study goes beyond the earlier studies by showing that nutrient uptake by phagocytosis can also allow the loss of genes involved in the synthesis of other organic compounds, such as coenzymes, sterols, and vitamins (fig. 3 and supplementary figs. S15–S17, Supplementary Material online).

### Prototrophic Host as a Potential Critical Component in the Evolution of Permanent Plastids?

Our analysis shows that two phago-mixotophic algae—the green alga *C. tetramitiformis* and the chlorarachniophyte *B. natans*—are similar to nonphagocytes in terms of their overall higher-level capacity to synthesize a range of small organic molecules compared with obligatory phagocytes and metazoans (fig. 3). This is in agreement with the fact that both algae are known to be able to grow in inorganic growth media, such as f/2 (Guillard and Ryther 1962) (which contains a B vitamin mixture). Many eukaryotic phototrophs can sustain their growth on inorganic substances (plus B vitamins); and although obligatorily phagotrophic algae do exist (e.g., the dinoflagellate *Dinophysis* and the euglenophyte *Rapaza*), their compulsory feeding is mostly likely a derived status as their close photosynthetic relatives are not obligatory phagotrophs (Hansen 2011; Yamaguchi et al. 2012). An interesting corollary thus is that heterotrophic eukaryotic ancestors that became photosynthetic through endosymbiosis likely had the ability to synthesize most, if not all, small organic molecules from inorganic compounds. Early eukaryotes indeed are predicted to be prototrophic (i.e., not requiring specific organic substances) as eukaryotes are believed to have originated from prokaryotes, which are bound by rigid cell wall and hence are required to be autonomous in terms of biosynthetic capacity (Cavalier-Smith 2002) unless adapted to extremely nutrient-rich environments (but see D’Souza et al. 2014). An irony is that nutritional dependence through the process of phagocytosis, which was an important process in the acquisition of plastids (Maruyama and Kim 2013), led to the loss of genes involved in small molecule synthesis in heterotrophic eukaryotes, which, in turn, might make it more difficult to permanently incorporate a photosynthetic endosymbiont. This might explain why evolution of eukaryotic photosynthesis in the “permanent” form has not occurred among metazoans, amoebozoans, or ciliates—all of which are auxotrophic for a number of amino acids and other small compounds (fig. 3; Payne and Loomis 2006)—despite many documented cases of their associations with photosynthetic endosymbionts/organelles (e.g., Wang and Douglas 1997; Rumpho et al. 2000; Wagele et al. 2011). Perhaps, there was a window of time (after eukaryogenesis) when eukaryotes were still mostly prototrophic and hence had a higher propensity to become phototrophs following endosymbiosis compared with more recent, mostly auxotrophic phagotrophs.

## Supplementary Material

Supplementary Data

Supplementary Data
