# Supplementary material for: Comparative Genomics of a Bacterivorous Green Alga Reveals Evolutionary Causalities and Consequences of Phago-Mixotrophic Mode of Nutrition
Source: Genome Biol Evol. 2015 Jul 29;7(11):3047–61. doi: 10.1093/gbe/evv144 (PMC5741210; doi:10.1093/gbe/evv144)

Species name	*Genome File*	*Peptide File*	*Resource*	*References*
Phagocytes				
Bigelowiella natans CCMP2755	Bigna1_nuclear_scaffolds .fasta.gz	Bigna1_filtered_proteins.fasta.gz	JGI	(Curtis, et al. 2012)
Dictyostelium discoideum	dicty_primary_genomic 21-Aug-2014	dicty_primary_protein.fasta 08-March-2014	dictybase.org	(Eichinger, et al. 2005)
Drosophila melanogaster	N/A	dmel-all-translation-r5.55.fasta	ftp.flybase.net	(Adams, et al. 2000; Pierre, et al. 2014)
Entamoeba histolytica	N/A	AmoebaDB-3.1_EhistolyticaHM1IMSS _AnnotatedProteins.fasta	amoebadb.org	(Aurrecoechea, et al. 2010; Loftus, et al. 2005)
Mus musculus	N/A	downloaded from http://www.uniprot.org/proteomes/UP000000589 on 08-March-2014	UniProtKB	(Chinwalla, et al. 2002; Magrane and Consortium 2011)
Tetrahymena thermophila	N/A	T_thermophila_oct2008_proteins.fasta	ciliate.org	(Eisen, et al. 2006)
Thecamonas trahens ATCC 50062	thecamonas_trahens_atcc_50062_1_supercontigs.fasta	thecamonas_trahens_atcc_50062_1_proteins.fasta 12-March-2014	Broad Institute	(Ruiz-Trillo, et al. 2007)
Non-phagocytes				
Arabidopsis thaliana	TAIR9_chr_all.fas	TAIR10_pep_20101214.fasta	TAIR	(Initiative 2000; Lamesch, et al. 2012)
Batrachochytrium dendrobatidis	N/A	*B. dendrobatidis* - proteins.fasta	Broad Institute	(MIT)
Chlamydomonas reinhardtii 281	Creinhardtii_281_v5.0.softmasked	Creinhardtii_236_protein.fa	Phytozyme	(Merchant, et al. 2007)
Cyanidioschyzon merolae	Complete Chromosomes 03-Jul-2007	cds.fasta 18-Jan-2008	merolae.biol.s.u-tokyo.ac.jp	(Matsuzaki, et al. 2004)
Oryza sativa	NA	Osativa_193_peptide.fa	Phytozome	(Ouyang, et al. 2007)
Saccharomyces cerevisiae strain S288C	N/A	orf_trans_all.fasta, 12-March-2014	Saccharomyces Genome Database (SGD)	(Cherry, et al. 2011; Engel, et al. 2014)
Other eukaryotes analyzed in this study				
Emiliania huxleyi	Emihu1_scaffolds.fa	N/A	JGI	(Read, et al. 2013)
Guillardia theta CCMP2712	Guith1_AssemblyScaffolds.fasta.gz	N/A	JGI	(Curtis, et al. 2012)

Homo sapiens	GCA_000001405.15_GRCh38_top-level.fna	N/A	GRC	(Lander, et al. 2001)
Micromonas pusilla C3	MicromonasCCMP1545.fasta.gz	MicpuC3_GeneCatalog_proteins_20110615.aa.fasta	JGI	(Worden, et al. 2009)
Monosiga brevicollis	Monbr1_scaffolds.fasta.gz	N/A	JGI	(King, et al. 2008)
Ostreococcus tauri	Otauri.fasta.gz	N/A	JGI	(Palenik, et al. 2007)
Physcomitrella patens	Physcomitrella_patens.1_1.fasta.gz	N/A	JGI	(Rensing, et al. 2008)
Thalassiosira pseudonana	Thaps3_chromosomes_assembly_chromosomes.fasta.gz	N/A	JGI	(Armbrust, et al. 2004; Bowler, et al. 2008)

Table S1. List of genome and protein files downloaded and used in this study.

Supplemental References

Adams MD, et al. 2000. The genome sequence of *Drosophila melanogaster*. Science 287:2185-2195.

Armbrust EV, et al. 2004. The genome of the diatom *Thalassiosira pseudonana*: ecology, evolution, and metabolism. Science 306:79-86.

Aurrecoechea C, et al. 2010. EuPathDB: a portal to eukaryotic pathogen databases. Nucleic Acids Res 38:D415-D419.

Bowler C, et al. 2008. The *Phaeodactylum* genome reveals the evolutionary history of diatom genomes. Nature 456:239-244.

Cherry JM, et al. 2011. *Saccharomyces* Genome Database: the genomics resource of budding yeast. Nucleic Acids Res. 40:D700-D705.

Chinwalla AT, et al. 2002. Initial sequencing and comparative analysis of the mouse genome. Nature 420:520-562.

Curtis BA, et al. 2012. Algal genomes reveal evolutionary mosaicism and the fate of nucleomorphs. Nature 492:59-65.

Eichinger L, et al. 2005. The genome of the social amoeba *Dictyostelium discoideum*. Nature 435:43-57.

Eisen JA, et al. 2006. Macronuclear genome sequence of the ciliate *Tetrahymena thermophila*, a model eukaryote. PLoS Biol. 4:e286.

Engel SR, et al. 2014. The reference genome sequence of *Saccharomyces cerevisiae*: then and now. G3: Genes| Genomes| Genetics 4:389-398.

Initiative AG 2000. Analysis of the genome sequence of the flowering plant *Arabidopsis thaliana*. Nature 408:796.

King N, et al. 2008. The genome of the choanoflagellate *Monosiga brevicollis* and the origin of metazoans. Nature 451:783-788.

Lamesch P, et al. 2012. The *Arabidopsis* Information Resource (TAIR): improved gene annotation and new tools. Nucleic Acids Res. 40:D1202-D1210.

Lander ES, et al. 2001. Initial sequencing and analysis of the human genome. Nature 409:860-921.

Loftus B, et al. 2005. The genome of the protist parasite *Entamoeba histolytica*. Nature 433:865-868.

Magrane M, Consortium U 2011. UniProt Knowledgebase: a hub of integrated protein

data. Database 2011: bar009.

Matsuzaki M, et al. 2004. Genome sequence of the ultrasmall unicellular red alga *Cyanidioschyzon merolae* 10D. Nature 428:653-657.

Merchant SS, et al. 2007. The *Chlamydomonas* genome reveals the evolution of key animal and plant functions. Science 318:245-250.

MIT BIoHa *Batrachochytrium dendrobatidis* Sequencing Project. http://www.broadinstitute.org/.

Ouyang S, et al. 2007. The TIGR rice genome annotation resource: improvements and new features. Nucleic Acids Res. 35:D883-D887.

Palenik B, et al. 2007. The tiny eukaryote *Ostreococcus* provides genomic insights into the paradox of plankton speciation. Proc Natl Acad Sci U S A. 104:7705-7710.

Pierre SES, Ponting L, Stefancsik R, McQuilton P 2014. FlyBase 102—advanced approaches to interrogating FlyBase. Nucleic Acids Res. 42:D780-D788.

Read BA, et al. 2013. Pan genome of the phytoplankton *Emiliania* underpins its global distribution. Nature 499: 209-213.

Rensing SA, et al. 2008. The *Physcomitrella* genome reveals evolutionary insights into the conquest of land by plants. Science 319:64-69.

Ruiz-Trillo I, et al. 2007. The origins of multicellularity: a multi-taxon genome initiative. Trends in Genetics 23:113-118.

Worden AZ, et al. 2009. Green evolution and dynamic adaptations revealed by genomes of the marine picoeukaryotes *Micromonas*. Science 324:268-272.

Supplemental Figure Legends

Figure S1. Kmer plot of reads prior to (A) and following (B) read filtering. Kmer-frequency (i.e., number of reads in each bin divided by the total number of reads) is plotted on the y-axis. Kmer depth (i.e., number of times a given kmer is represented in the genome) is plotted on the x-axis. Each bin is colored by the GC percentage from all of the kmers in that bin. The black arrowhead indicates the position of a highly represented, low GC content peak corresponding to sequencing reads that were mapped to organellar sequences.

Figure S2. Proportion of enriched peptide sets with homologs in known phagosome. A. *C. tetramitiformis* peptides enriched in phagocytes. B. *C. tetramitiformis* peptides enriched among non-phagocytes.

Figure S3. **(**A) Total number of *C. tetramitiformis* peptides in enrichment sets. The red dashed line illustrates 30 peptides, which we set as the lower limit for this analysis. (B) Curve illustrating the penalty assigned to each set size. The optimum is set at 4,305 peptides, which is the total number of *C. Tetramitiformis* genes that show significant homology to known phagosome components, and hence would receive a penalty of 0. Any gene set with greater or fewer peptides than this number receives the penalty defined by this curve.

Figure S4. (A) Weighted scores of sets of *C. tetramitiformis* peptides enriched in phagocytes. (B) Weighted scores of sets of *C. tetramitiformis* peptides enriched in

phagocytes plotted with the corresponding sets enriched in non-phagocytes. The red vertical arrows illustrate the distance between the two curves that was used to compute the distance score.

Figure S5. Distance scores for sets of *C. tetramitiformis* peptides enriched among phagocytes compared to those enriched among non-phagocytes. The red arrowhead indicates the clear peak indicating the set with the best balance of sensitivity and specificity (present in any 6 or more phagocytes, absent from any 3 or more non-phagocytes) that has the most peptides that is enriched in known phagosome components and is very distant from the corresponding set of *C. tetramitiformis* peptides enriched in non-phagocytes.

Figure S6. (A) Per base coverage of the *C. tetramitiformis* genome assembly. The dashed red line indicates the modal per base coverage of 19. Locations with 0 coverage are strings of N's where contigs were spliced together using the jumping library. (B) Average coverage per scaffold. The dashed red line indicates the modal value of 21.

Figure S7. GH103 ML tree.

Figure S8. GH28 ML tree.

Figure S9. GT51 ML tree.

Figure S10. Glucoamylase ML tree.

Figure S11. 4-Hydroxybutyryl-CoA dehydratase ML tree.

Figure S12. Biological Processes GO terms for *C. tetramitiformis* peptides enriched in phagocytes.

Figure S13. Cellular Component GO terms for *C. tetramitiformis* peptides enriched in phagocytes.

Figure S14. Molecular Function GO terms for *C. tetramitiformis* peptides enriched in phagocytes.

Figure S15. Biological Processes GO terms for *C. tetramitiformis* peptides enriched in non-phagocytes.

Figure S16. Cellular Component GO terms for *C. tetramitiformis* peptides enriched in non-phagocytes.

Figure S17. Molecular Function GO terms for *C. tetramitiformis* peptides enriched in non-phagocytes.

Figure S1

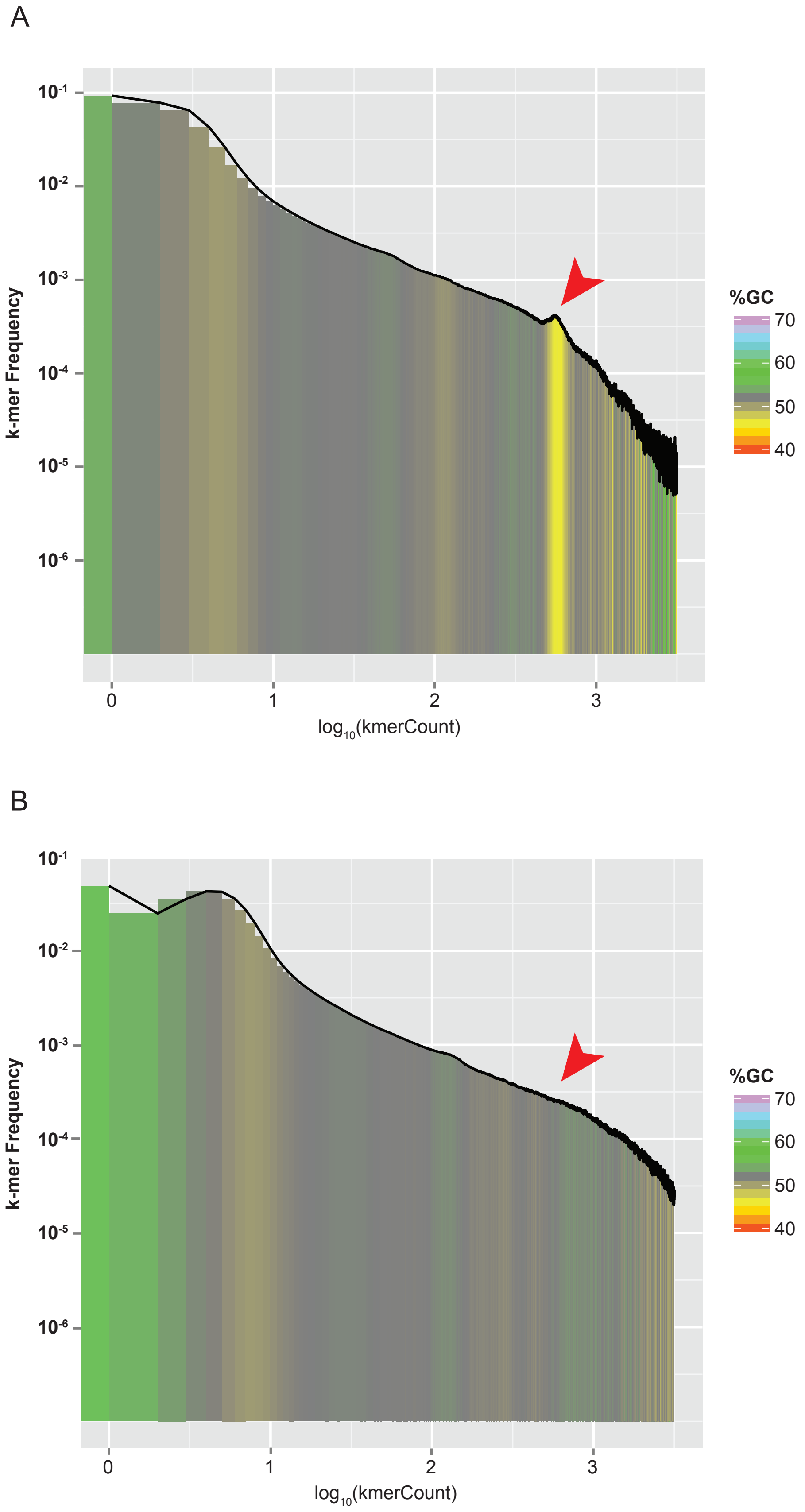
A
k-mer Frequency
10^{-1}
10^{-2}
10^{-3}
10^{-4}
10^{-5}
10^{-6}
0
1
2
3
log_{10}(kmerCount)
%GC
70
60
50
40
B
k-mer Frequency
10^{-1}
10^{-2}
10^{-3}
10^{-4}
10^{-5}
10^{-6}
0
1
2
3
log_{10}(kmerCount)
%GC
70
60
50
40

Figure S2

A

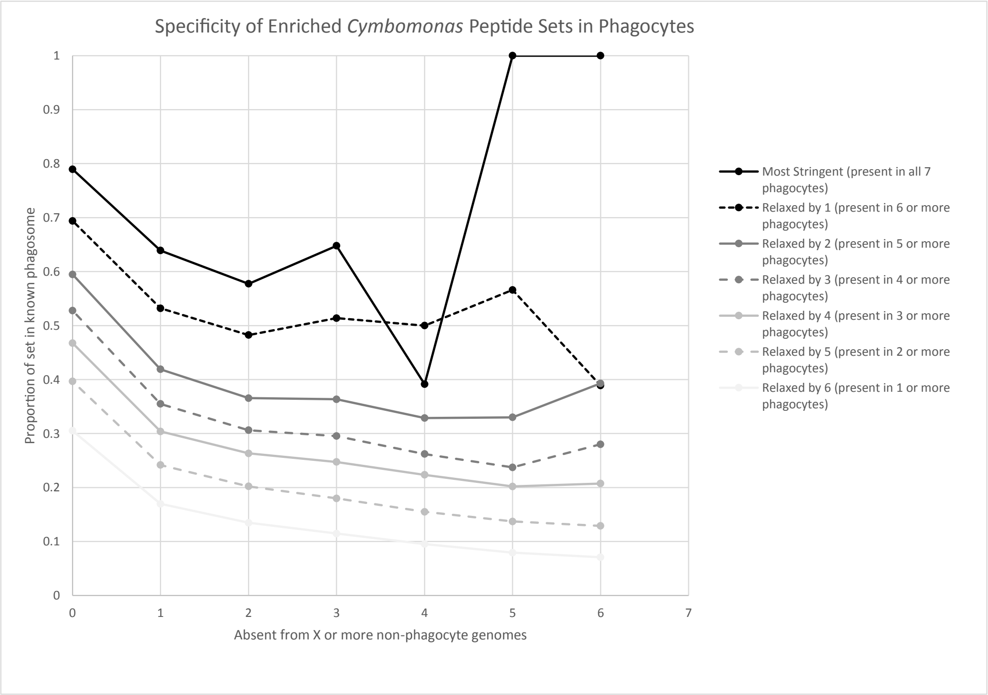

Specificity of Enriched *Cymbomonas* Peptide Sets in Phagocytes
Proportion of set in known phagosome
Absent from X or more non-phagocyte genomes
Most Stringent (present in all 7 phagocytes)
Relaxed by 1 (present in 6 or more phagocytes)
Relaxed by 2 (present in 5 or more phagocytes)
Relaxed by 3 (present in 4 or more phagocytes)
Relaxed by 4 (present in 3 or more phagocytes)
Relaxed by 5 (present in 2 or more phagocytes)
Relaxed by 6 (present in 1 or more phagocytes)

B

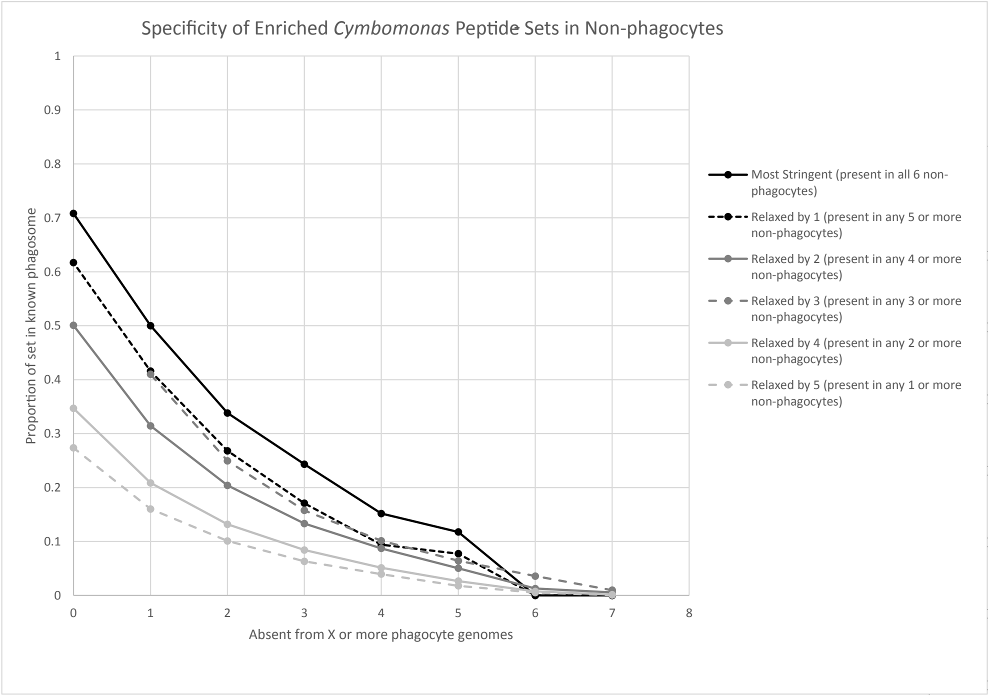

Specificity of Enriched *Cymbomonas* Peptide Sets in Non-phagocytes
Proportion of set in known phagosome
Absent from X or more phagocyte genomes
Most Stringent (present in all 6 non-phagocytes)
Relaxed by 1 (present in any 5 or more non-phagocytes)
Relaxed by 2 (present in any 4 or more non-phagocytes)
Relaxed by 3 (present in any 3 or more non-phagocytes)
Relaxed by 4 (present in any 2 or more non-phagocytes)
Relaxed by 5 (present in any 1 or more non-phagocytes)

Figure S3

A

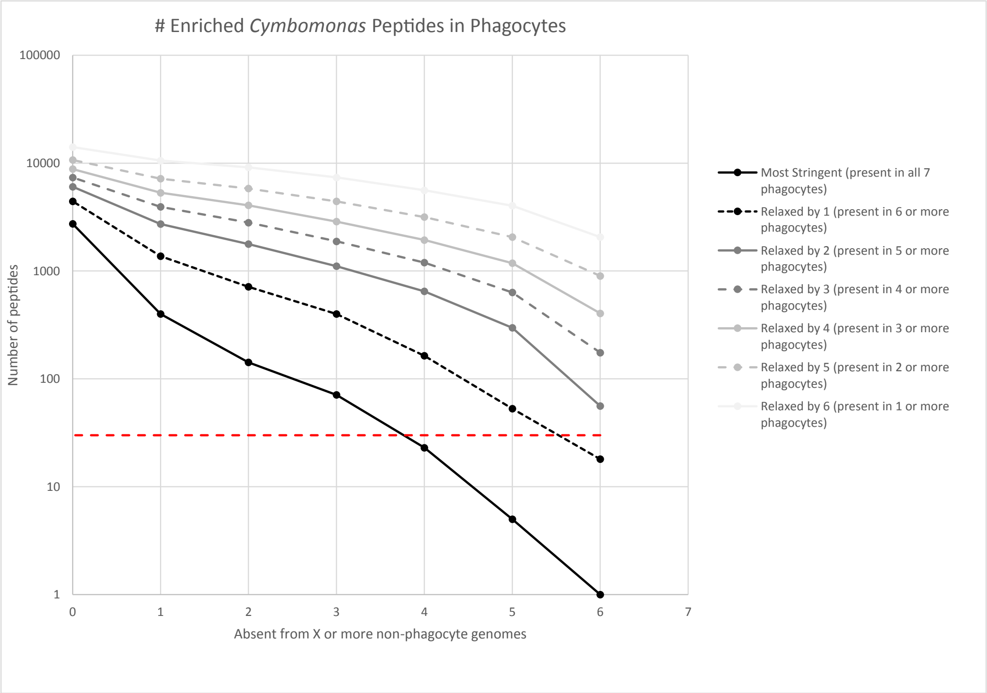

Enriched *Cymbomonas* Peptides in Phagocytes
100000
10000
1000
100
10
1
Number of peptides
0
1
2
3
4
5
6
7
Absent from X or more non-phagocyte genomes
Most Stringent (present in all 7 phagocytes)
Relaxed by 1 (present in 6 or more phagocytes)
Relaxed by 2 (present in 5 or more phagocytes)
Relaxed by 3 (present in 4 or more phagocytes)
Relaxed by 4 (present in 3 or more phagocytes)
Relaxed by 5 (present in 2 or more phagocytes)
Relaxed by 6 (present in 1 or more phagocytes)

B

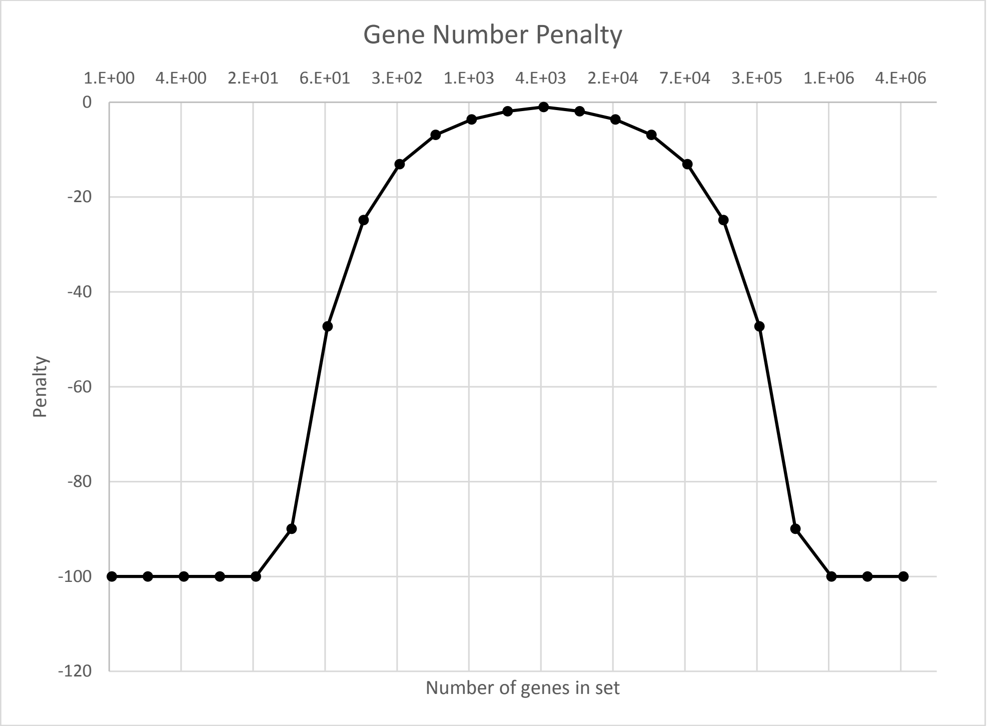

Gene Number Penalty
1.E+00
4.E+00
2.E+01
6.E+01
3.E+02
1.E+03
4.E+03
2.E+04
7.E+04
3.E+05
1.E+06
4.E+06
0
-20
-40
-60
-80
-100
-120
Penalty
Number of genes in set

Figure S4

A

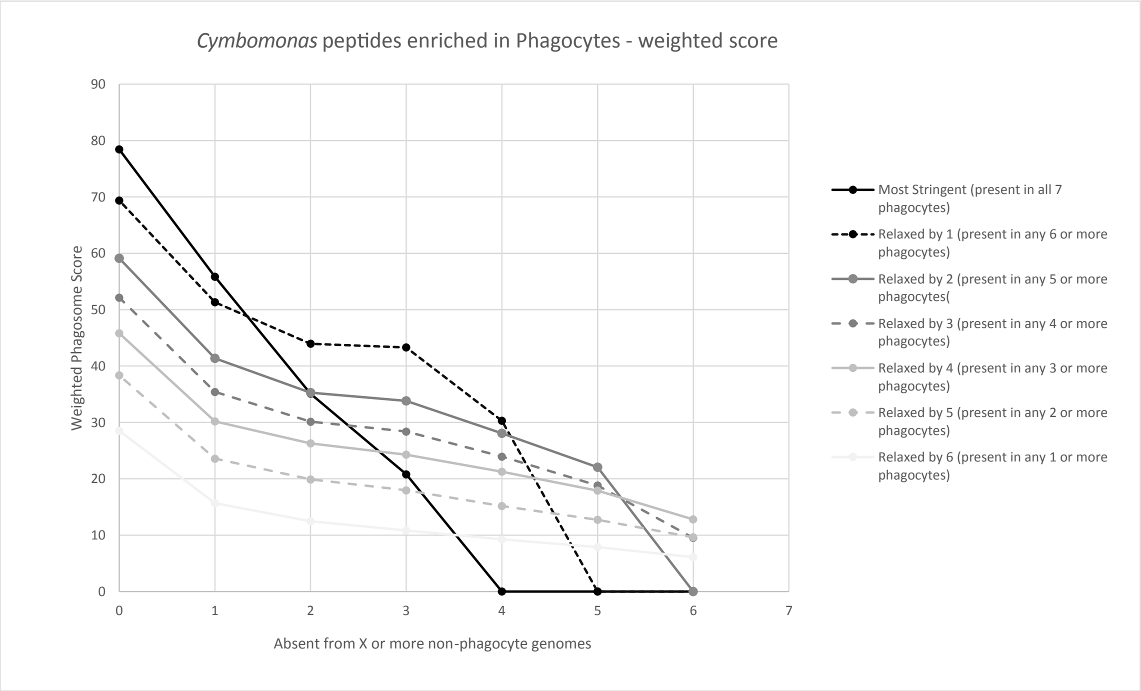
Cymbomonas peptides enriched in Phagocytes - weighted score
Weighted Phagosome Score
0
10
20
30
40
50
60
70
80
90
0
1
2
3
4
5
6
7
Absent from X or more non-phagocyte genomes
Most Stringent (present in all 7 phagocytes)
Relaxed by 1 (present in any 6 or more phagocytes)
Relaxed by 2 (present in any 5 or more phagocytes(
Relaxed by 3 (present in any 4 or more phagocytes)
Relaxed by 4 (present in any 3 or more phagocytes)
Relaxed by 5 (present in any 2 or more phagocytes)
Relaxed by 6 (present in any 1 or more phagocytes)

B

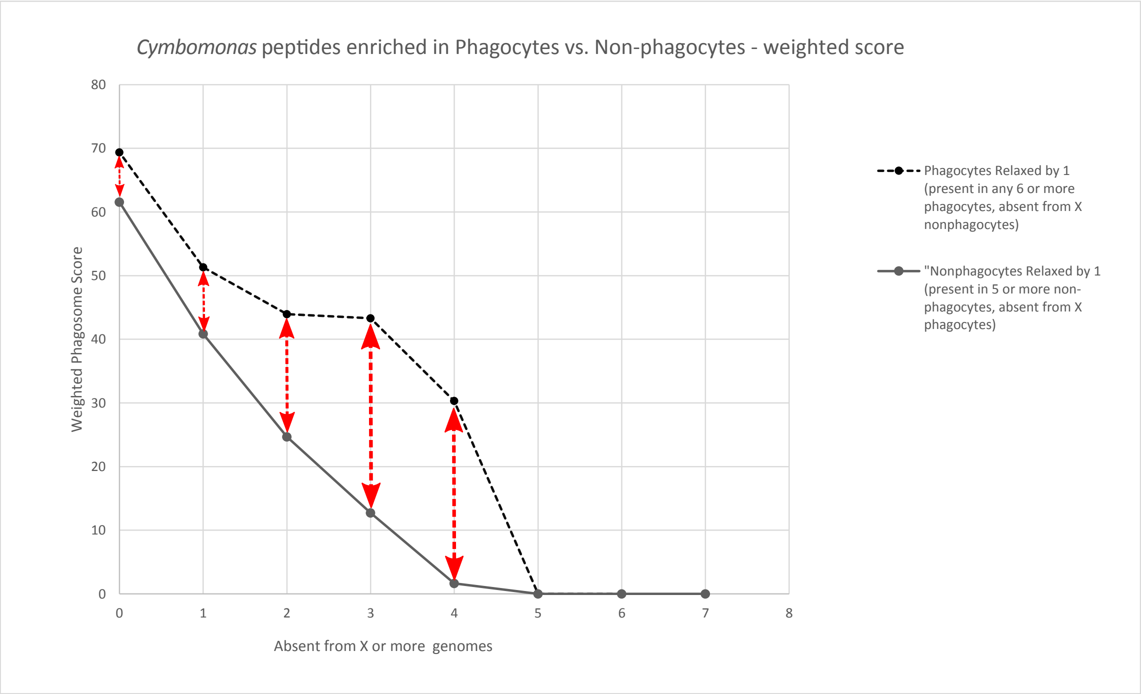
Cymbomonas peptides enriched in Phagocytes vs. Non-phagocytes - weighted score
Weighted Phagosome Score
0
10
20
30
40
50
60
70
80
0
1
2
3
4
5
6
7
8
Absent from X or more genomes
Phagocytes Relaxed by 1 (present in any 6 or more phagocytes, absent from X nonphagocytes)
"Nonphagocytes Relaxed by 1 (present in 5 or more non-phagocytes, absent from X phagocytes)

Figure S5

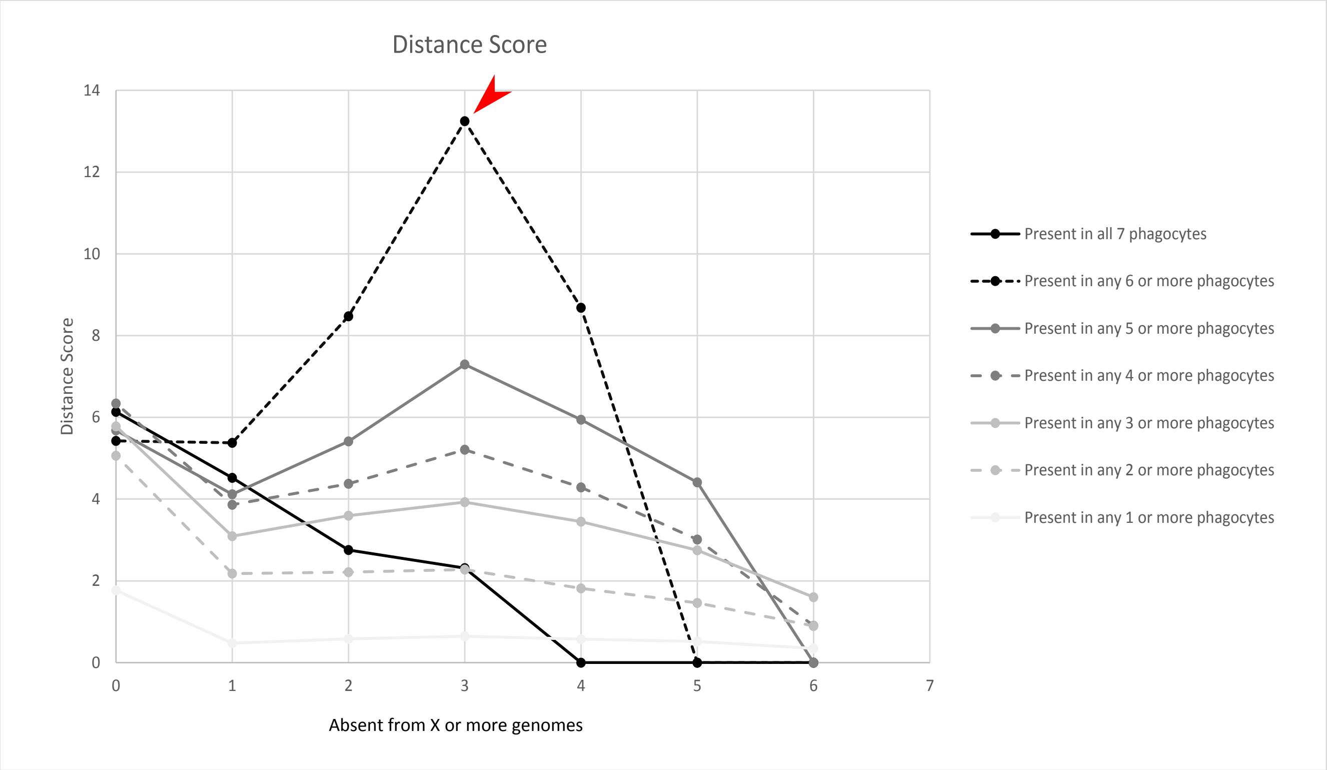
Distance Score
14
12
10
8
6
4
2
0
Distance Score
0
1
2
3
4
5
6
7
Absent from X or more genomes
Present in all 7 phagocytes
Present in any 6 or more phagocytes
Present in any 5 or more phagocytes
Present in any 4 or more phagocytes
Present in any 3 or more phagocytes
Present in any 2 or more phagocytes
Present in any 1 or more phagocytes

Figure S6

A.

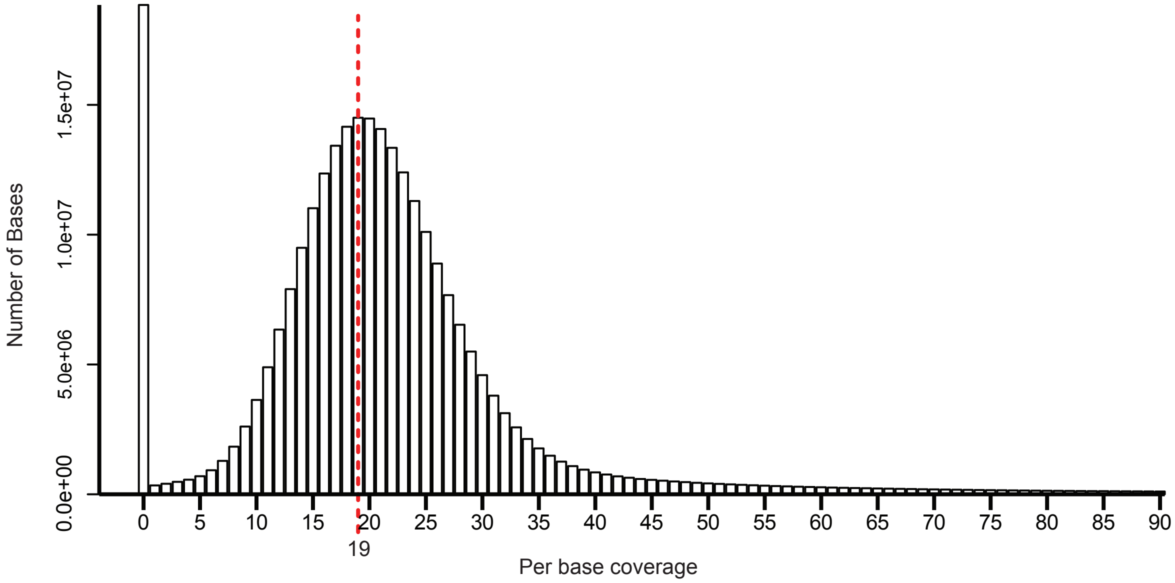

Number of Bases
0.0e+00
5.0e+06
1.0e+07
1.5e+07
0 5 10 15 20 25 30 35 40 45 50 55 60 65 70 75 80 85 90
19
Per base coverage

B.

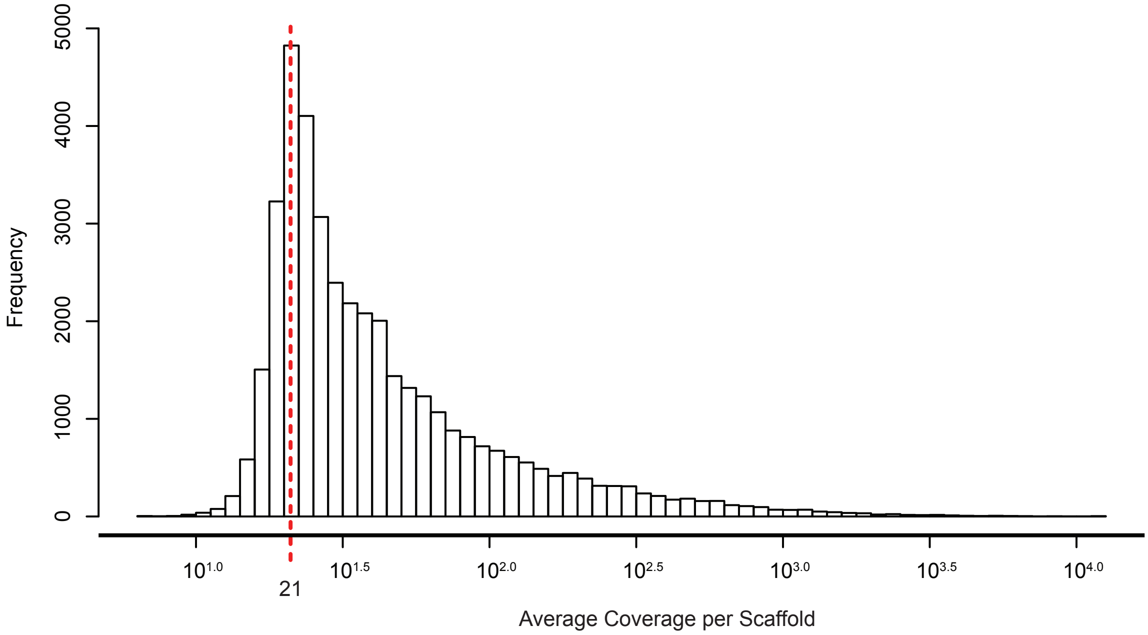

Frequency
0
1000
2000
3000
4000
5000
$10^{1.0}$ $10^{1.5}$ $10^{2.0}$ $10^{2.5}$ $10^{3.0}$ $10^{3.5}$ $10^{4.0}$
21
Average Coverage per Scaffold

Figure S7. GH103 ML tree

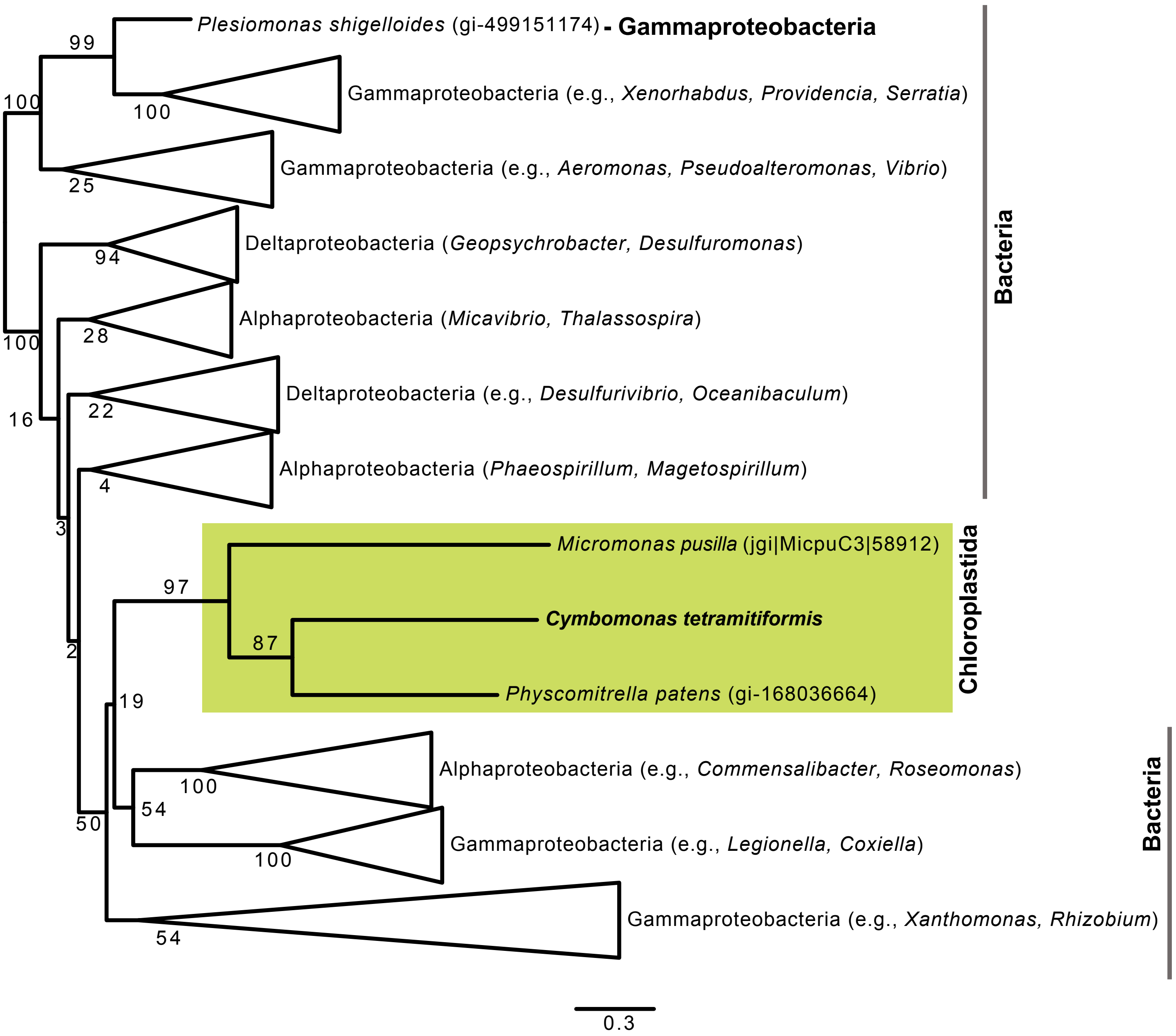

Figure S8. GH28 ML tree

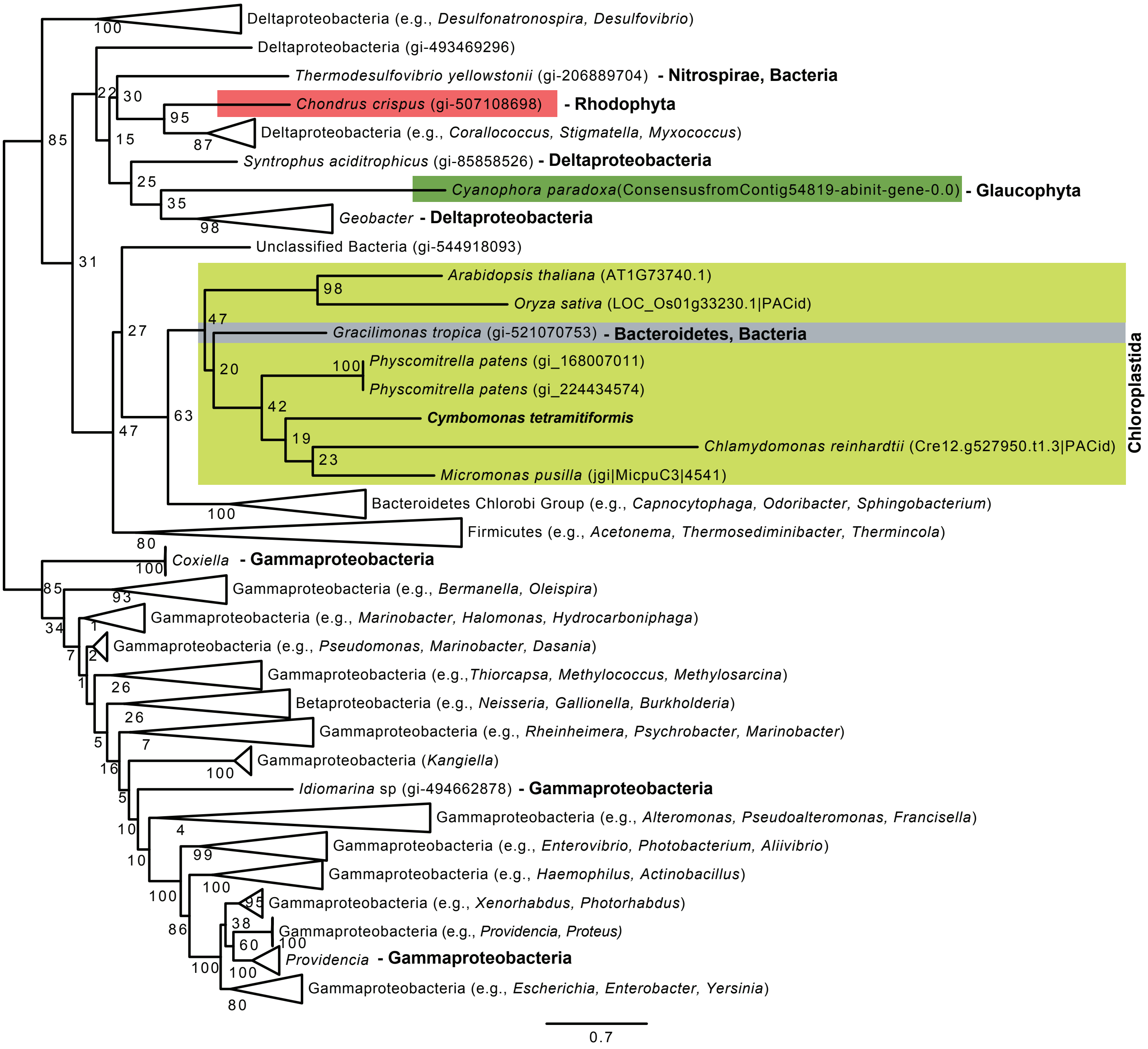

Figure S9. GT51 ML tree

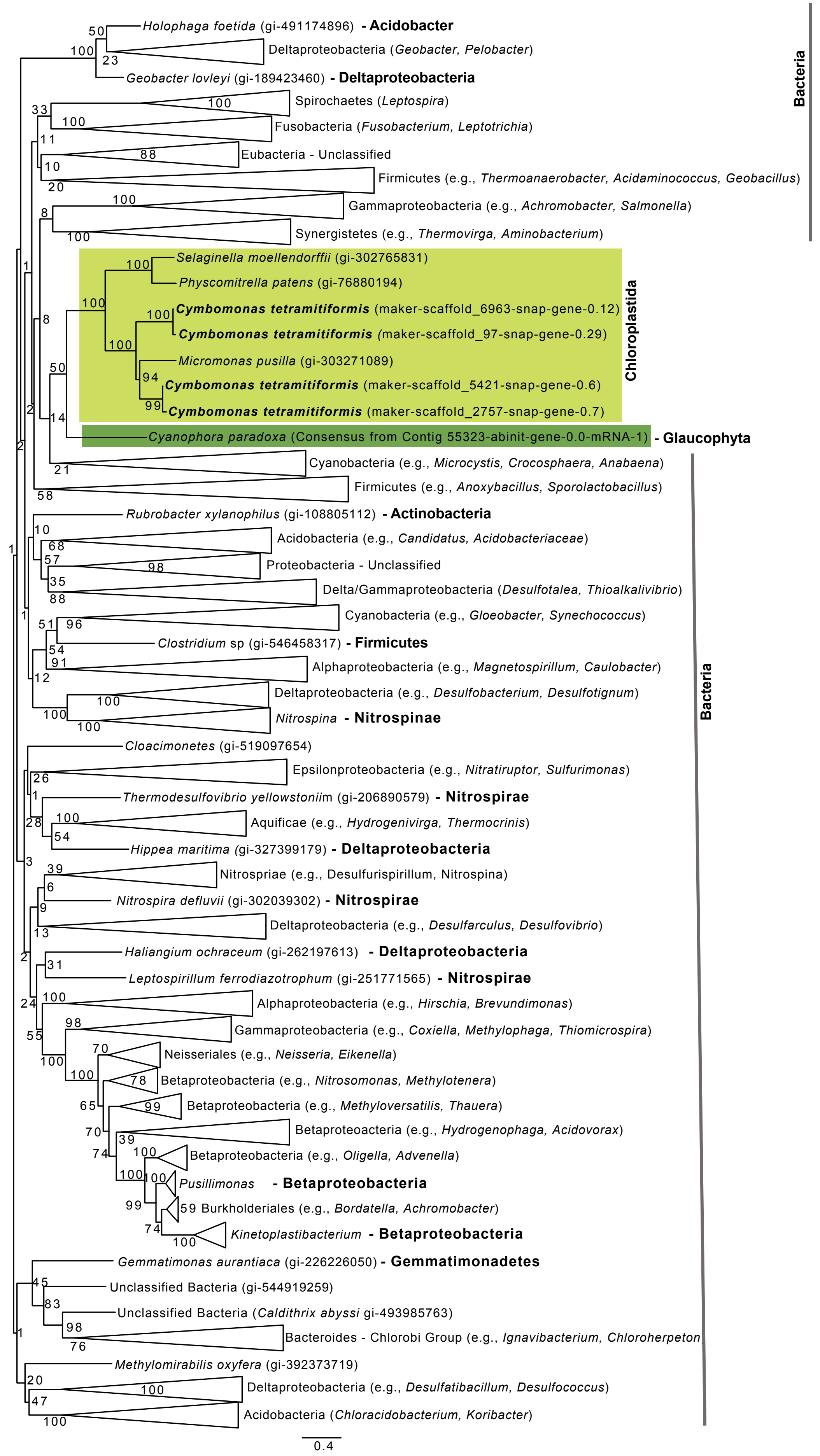

Figure S10. Glucoamylase ML tree

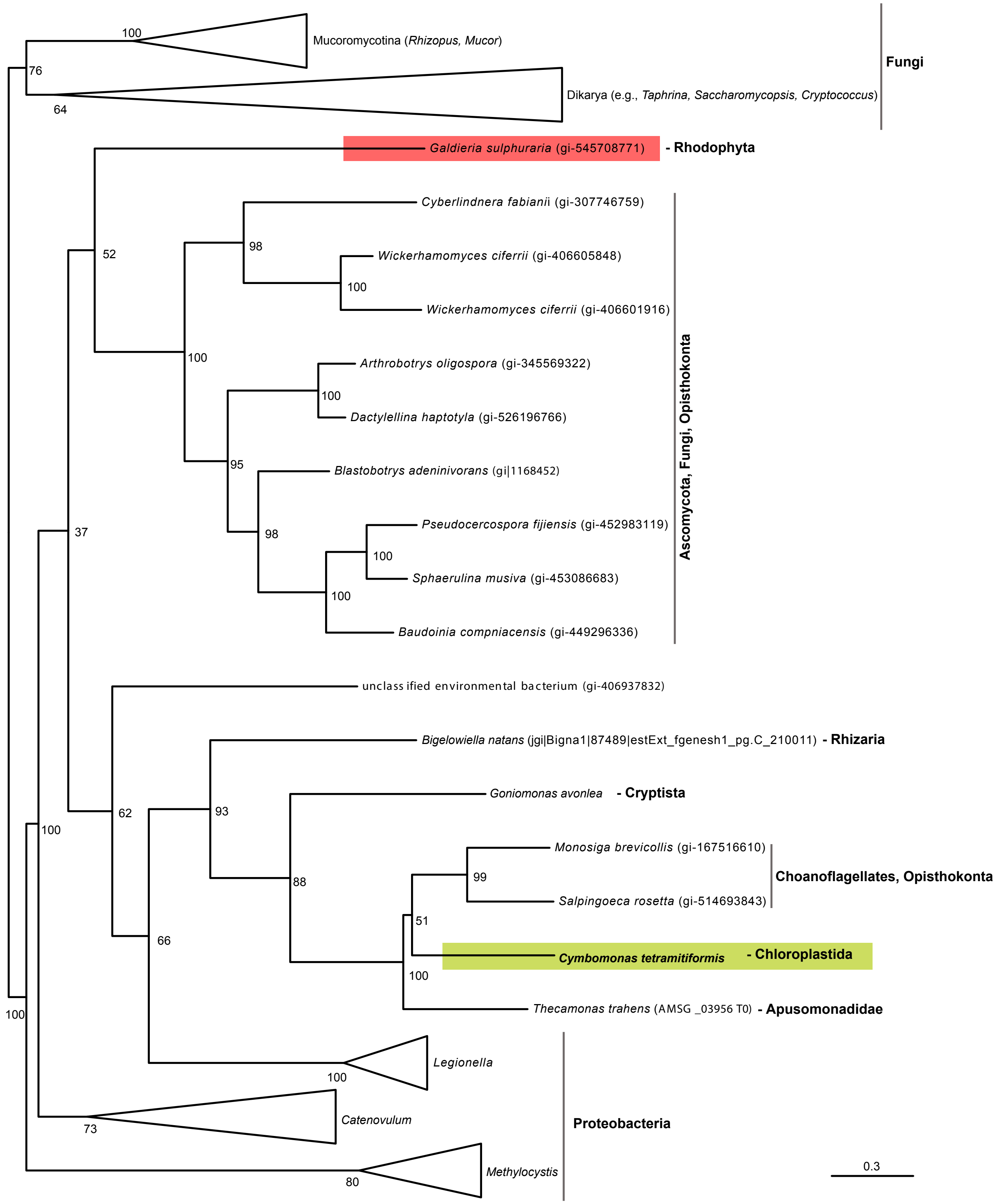

Figure S11. 4-Hydroxybutyryl-CoA dehydratase ML tree

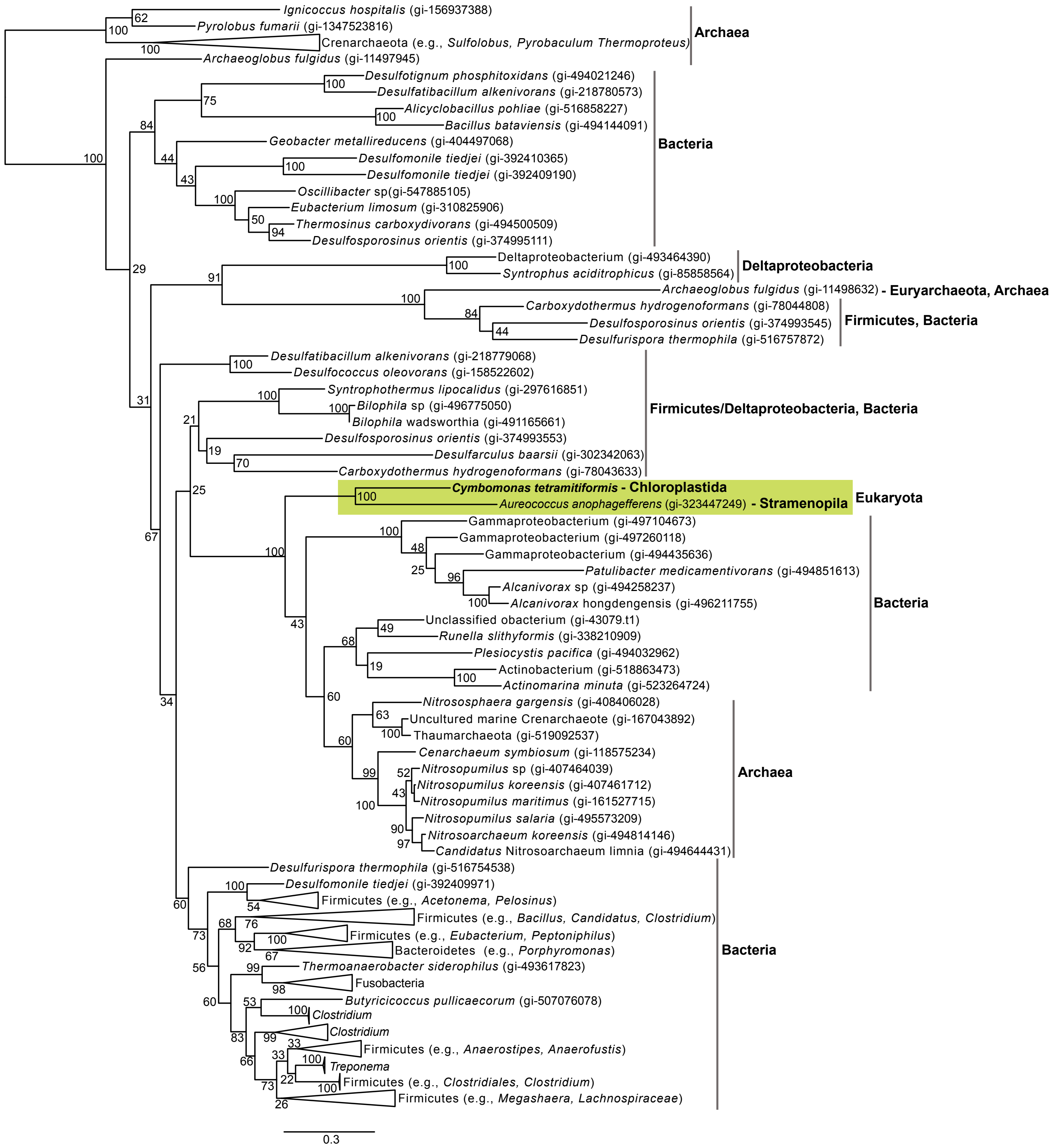

Figure S12. Biological Process GO terms for *C. tetramitiformis* peptides enriched in phagocytes

GO.ID	Term-Biological Process	# C.tet. peptides in GO category	# peptides in phagocyte enrichment	GO enrichment p-value	Phagocytes score	Non-phagocytes score
GO:0044351	macropinocytosis	104	10	0.0081	6.20	1.30
GO:0051930	regulation of sensory perception of pain	4	3	0.0002	6.00	1.33
GO:0006898	receptor-mediated endocytosis	19	4	0.0059	6.20	1.40
GO:0030036	actin cytoskeleton organization	101	10	0.0067	6.27	1.64
GO:0046068	cGMP metabolic process	12	3	0.0105	6.00	1.67
GO:0051258	protein polymerization	63	6	0.0385	6.43	1.71
GO:0006013	mannose metabolic process	4	3	0.0002	6.00	2.00
GO:0051260	protein homooligomerization	57	8	0.0017	6.46	2.00
GO:0050804	regulation of synaptic transmission	21	4	0.0086	6.00	2.00
GO:0030587	sorocarp development	36	5	0.0132	6.33	2.00
GO:0030198	extracellular matrix organization	36	5	0.0132	6.00	2.00
GO:0008203	cholesterol metabolic process	24	4	0.0139	6.40	2.00
GO:0003008	system process	190	21	0.0090	6.25	2.13
GO:0007399	nervous system development	251	20	0.0086	6.05	2.14
GO:0018095	protein polyglutamylation	12	5	0.0001	6.00	2.17
GO:0051291	protein heterooligomerization	11	3	0.0081	6.60	2.20
GO:0007155	cell adhesion	140	23	0.0000	6.15	2.21
GO:0022610	biological adhesion	141	24	0.0368	6.15	2.24
GO:0035249	synaptic transmission, glutamatergic	11	3	0.0081	6.00	2.25
GO:0016265	death	217	15	0.0259	6.20	2.27
GO:0030030	cell projection organization	194	21	0.0327	6.08	2.28
GO:0052652	cyclic purine nucleotide metabolic process	17	4	0.0039	6.33	2.33
GO:0050803	regulation of synapse structure or activity	12	3	0.0105	6.00	2.33
GO:0031122	cytoplasmic microtubule organization	18	3	0.0328	6.00	2.33
GO:1900542	regulation of purine nucleotide metabolic process	111	9	0.0323	6.18	2.36
GO:0009150	purine ribonucleotide metabolic process	596	35	0.0362	6.06	2.40
GO:0007626	locomotory behavior	33	5	0.0092	6.20	2.40
GO:0070848	response to growth factor	42	5	0.0246	6.00	2.40
GO:0072523	purine-containing compound catabolic process	529	32	0.0353	6.02	2.44
GO:1901069	guanosine-containing compound catabolic process	148	11	0.0335	6.00	2.45
GO:0023052	signaling	837	52	0.0111	6.17	2.47
GO:0007154	cell communication	889	53	0.0370	6.19	2.47
GO:0019934	cGMP-mediated signaling	4	4	0.0000	6.00	2.50
GO:0009611	response to wounding	87	11	0.0006	6.08	2.50
GO:0008015	blood circulation	43	6	0.0067	6.63	2.50
GO:0051262	protein tetramerization	21	4	0.0086	6.25	2.50
GO:0006954	inflammatory response	31	4	0.0332	6.25	2.50
GO:0018958	phenol-containing compound metabolic process	32	4	0.0368	6.50	2.50
GO:0055002	striated muscle cell development	19	3	0.0378	6.75	2.50
GO:0007018	microtubule-based movement	120	23	0.0000	6.00	2.57
GO:0071900	regulation of protein serine/threonine kinase activity	58	6	0.0270	6.29	2.57
GO:0006198	cAMP catabolic process	7	3	0.0019	6.00	2.67
GO:0007041	lysosomal transport	10	3	0.0061	6.33	2.67
GO:0090130	tissue migration	16	3	0.0238	6.00	2.67
GO:0035082	axoneme assembly	16	5	0.0003	6.00	2.71
GO:0048729	tissue morphogenesis	66	9	0.0277	6.36	2.73
GO:0050808	synapse organization	27	5	0.0038	6.20	2.80
GO:0042493	response to drug	63	6	0.0385	6.50	2.83
GO:0006508	proteolysis	435	31	0.0054	6.40	2.87
GO:2000014	regulation of endosperm development	3	3	0.0001	6.17	3.00
GO:0060441	epithelial tube branching involved in lung morphogenesis	7	3	0.0019	6.80	3.00
GO:0008206	bile acid metabolic process	9	3	0.0044	6.33	3.00

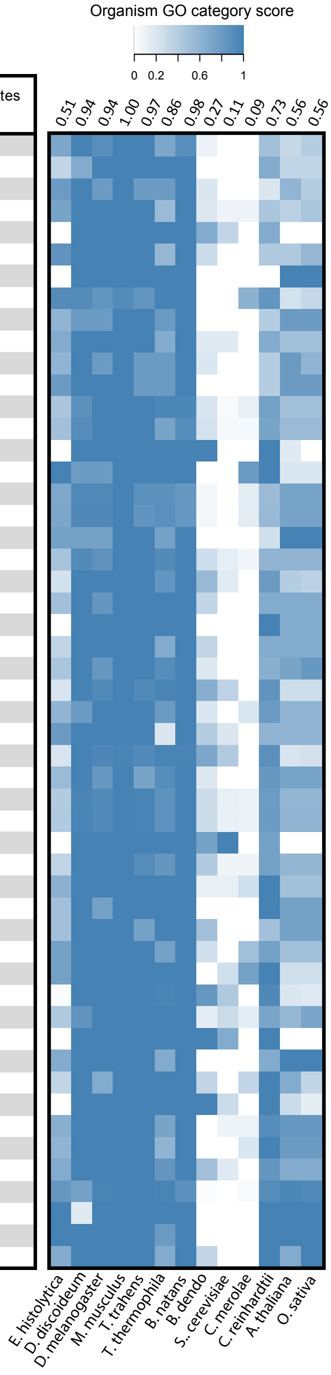

Figure S13. Cellular Compartment GO terms for *C. tetramitiformis* peptides enriched in phagocytes

GO.ID	Term-Cellular Compartment	# C.tet. peptides in GO category	# peptides in phagocyte enrichment	GO enrichment p-value	Phagocytes score	Non-phagocytes score
GO:0071203	WASH complex	6	3	0.0013	6.67	0.33
GO:0030016	myofibril	30	3	0.1246	6.33	1.33
GO:0034703	cation channel complex	70	8	0.0077	6.25	1.75
GO:0005938	cell cortex	63	3	0.4852	6.50	1.75
GO:0016323	basolateral plasma membrane	35	5	0.0135	6.14	1.86
GO:0019898	extrinsic component of membrane	34	4	0.0496	6.43	1.86
GO:0005604	basement membrane	26	7	0.0001	6.00	2.00
GO:0009986	cell surface	67	12	0.0006	6.42	2.05
GO:0005576	extracellular region	512	59	0.0001	6.33	2.13
GO:0005768	endosome	167	14	0.0087	6.37	2.16
GO:0044459	plasma membrane part	307	33	0.0762	6.30	2.16
GO:0070062	extracellular vesicular exosome	241	26	0.0000	6.32	2.16
GO:0031982	vesicle	418	41	0.1410	6.31	2.19
GO:0005615	extracellular space	113	21	0.0000	6.43	2.23
GO:0016023	cytoplasmic membrane-bounded vesicle	190	16	0.0049	6.42	2.26
GO:0005886	plasma membrane	789	47	0.5666	6.28	2.28
GO:0005764	lysosome	105	21	0.0000	6.57	2.29
GO:0043005	neuron projection	117	14	0.0003	6.00	2.31
GO:0005887	integral component of plasma membrane	134	19	0.0000	6.33	2.33
GO:0005925	focal adhesion	11	3	0.0089	6.00	2.33
GO:0071944	cell periphery	870	50	0.5711	6.28	2.34
GO:0097458	neuron part	144	17	0.0891	6.00	2.37
GO:0005856	cytoskeleton	493	54	0.0097	6.01	2.45
GO:0005829	cytosol	680	28	0.5356	6.22	2.50
GO:0005929	cilium	156	26	0.0001	6.00	2.53
GO:0030054	cell junction	169	12	0.1953	6.08	2.54
GO:0048471	perinuclear region of cytoplasm	94	8	0.0393	6.27	2.55
GO:0045202	synapse	73	9	0.0423	6.10	2.60
GO:0044463	cell projection part	149	22	0.0058	6.00	2.65
GO:0036477	somatodendritic compartment	89	8	0.0296	6.00	2.67
GO:0005635	nuclear envelope	80	5	0.2336	6.33	2.67
GO:0000151	ubiquitin ligase complex	59	3	0.4420	6.00	2.67
GO:0005874	microtubule	181	32	0.0000	6.02	2.70
GO:0031594	neuromuscular junction	12	3	0.0116	6.00	3.00
GO:0043025	neuronal cell body	56	6	0.0268	6.00	3.00
GO:0005618	cell wall	62	3	0.4745	6.20	3.00

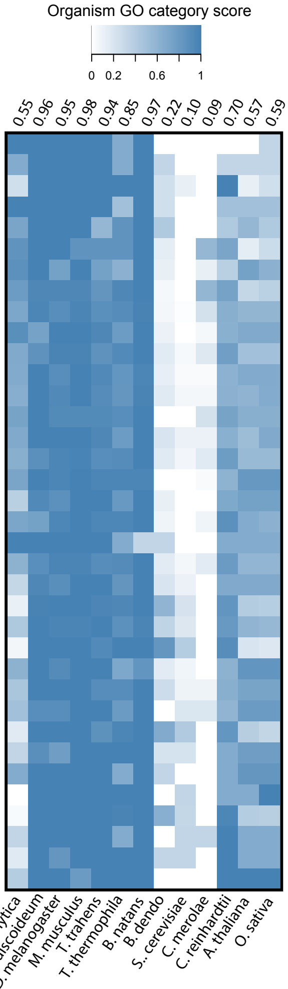

Figure S14. Molecular Function GO terms for *C. tetramitiformis* peptides enriched in phagocytes

GO.ID	Term-Molecular Function	# C.tet. peptides in GO category	# peptides in phagocyte enrichment	GO enrichment p-value	Phagocytes score	Non-phagocytes score
GO:0008441	3'(2'),5'-bisphosphate nucleotidase activity	4	3	0.0003	6.00	0.50
GO:0004620	phospholipase activity	16	3	0.0259	6.67	1.33
GO:0003779	actin binding	97	11	0.0020	6.00	1.36
GO:0015077	monovalent inorganic cation transmemb. transporter	143	9	0.1341	6.22	1.56
GO:0022843	voltage-gated cation channel activity	104	8	0.0638	6.13	1.63
GO:0005178	integrin binding	9	3	0.0048	6.00	1.67
GO:0005261	cation channel activity	135	9	0.1035	6.22	1.78
GO:0022890	inorganic cation transmembrane transporter activity	226	12	0.2200	6.25	1.83
GO:0004559	alpha-mannosidase activity	10	4	0.0005	6.00	2.00
GO:0030246	carbohydrate binding	66	8	0.0053	6.00	2.00
GO:0042802	identical protein binding	186	15	0.0093	6.17	2.00
GO:0004930	G-protein coupled receptor activity	19	3	0.0410	6.25	2.00
GO:0005509	calcium ion binding	225	33	0.0000	6.30	2.06
GO:0004872	receptor activity	114	11	0.0605	6.36	2.14
GO:0008201	heparin binding	23	6	0.0003	6.27	2.18
GO:0046872	metal ion binding	1534	87	0.1813	6.18	2.21
GO:0030551	cyclic nucleotide binding	28	6	0.1166	6.00	2.33
GO:0008289	lipid binding	92	10	0.0043	6.27	2.36
GO:0016810	hydrolase activity, on C-N (not peptide) bonds	61	5	0.1050	6.40	2.40
GO:0047555	3',5'-cyclic-GMP phosphodiesterase activity	9	5	0.0000	6.00	2.60
GO:0030552	cAMP binding	25	5	0.0031	6.00	2.60
GO:0004115	3',5'-cyclic-AMP phosphodiesterase activity	4	3	0.0003	6.00	2.67
GO:0008236	serine-type peptidase activity	56	7	0.0076	6.67	2.67
GO:0005518	collagen binding	11	3	0.0089	6.33	2.67
GO:0005096	GTPase activator activity	42	3	0.2486	6.00	2.67
GO:0003777	microtubule motor activity	91	26	0.0000	6.00	2.70
GO:0008158	hedgehog receptor activity	10	3	0.0067	6.50	2.75
GO:0070736	protein-GLY ligase activity, initiating	3	3	0.0001	6.00	2.80
GO:0004175	endopeptidase activity	119	15	0.0336	6.21	2.88
GO:0008234	cysteine-type peptidase activity	63	14	0.0014	6.48	2.88
GO:0008233	peptidase activity	237	27	0.1723	6.44	2.91
GO:0004198	Ca-dependent CYS-type endopeptidase activity	8	6	0.0000	6.08	3.00
GO:0008238	exopeptidase activity	52	4	0.1648	6.50	3.00

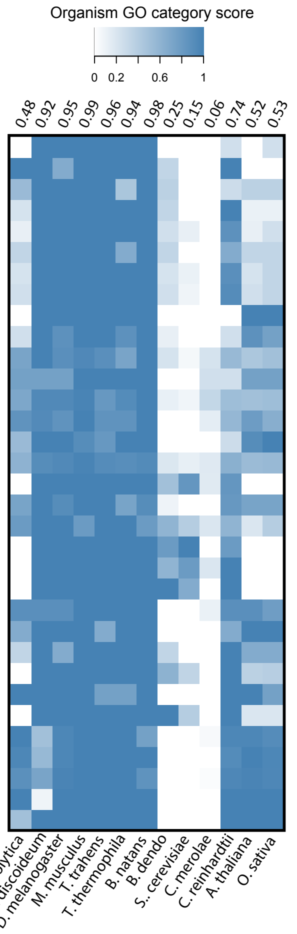

Figure S15. Biological Processes GO terms for *C. tetramitiformis* peptides enriched in non-phagocytes

GO.ID	Term-Biological Process	# C.tet. peptides in GO category	# peptides in non-phagocyte enrichment	GO enrichment p-value	Phagocytes score	Non-phagocytes score
GO:0000162	tryptophan biosynthetic process	5	4	0.0001	0.75	5.75
GO:0009082	branched-chain amino acid biosynthetic process	15	4	0.0182	1.00	5.25
GO:0046417	chorismate metabolic process	6	5	0.0050	1.20	5.60
GO:0009423	chorismate biosynthetic process	4	3	0.0013	1.33	5.33
GO:0043650	dicarboxylic acid biosynthetic process	12	6	0.0149	1.50	5.50
GO:0009073	aromatic amino acid family biosynthetic process	29	10	0.0053	1.60	5.60
GO:0009081	branched-chain amino acid metabolic process	26	5	0.0334	1.60	5.40
GO:0009088	threonine biosynthetic process	6	5	0.0000	1.67	5.67
GO:0016925	protein sumoylation	11	3	0.0381	1.67	5.67
GO:0000105	histidine biosynthetic process	6	5	0.0000	2.00	6.00
GO:0048653	anther development	9	3	0.0216	2.00	5.33
GO:0042364	water-soluble vitamin biosynthetic process	30	10	0.0005	2.10	5.70
GO:0043604	amide biosynthetic process	14	5	0.0477	2.20	6.00
GO:0009110	vitamin biosynthetic process	36	12	0.0601	2.25	5.67
GO:0016132	brassinosteroid biosynthetic process	10	5	0.0003	2.40	5.60
GO:0006526	arginine biosynthetic process	7	4	0.0007	2.50	5.50
GO:0006646	phosphatidylethanolamine biosynthetic process	6	3	0.0060	2.50	5.00
GO:0009108	coenzyme biosynthetic process	77	12	0.0369	2.50	5.75
GO:0016126	sterol biosynthetic process	34	11	0.0000	2.53	5.33
GO:1901617	organic hydroxy compound biosynthetic process	97	25	0.0048	2.61	5.32
GO:0048316	seed development	96	15	0.0029	2.63	5.11
GO:0001522	pseudouridine synthesis	24	6	0.0055	2.67	5.33
GO:0042631	cellular response to water deprivation	13	3	0.0594	2.67	5.00
GO:0044711	single-organism biosynthetic process	889	113	0.0697	2.69	5.39
GO:0009064	glutamine family amino acid metabolic process	40	14	0.0038	2.71	5.79
GO:0006084	acetyl-CoA metabolic process	18	5	0.0069	2.80	5.60
GO:0046474	glycerophospholipid biosynthetic process	78	12	0.0647	2.85	5.08
GO:0006863	purine nucleobase transport	5	3	0.0032	3.00	5.00
GO:0072330	monocarboxylic acid biosynthetic process	123	14	0.0520	3.00	5.43
GO:0048527	lateral root development	13	3	0.0594	3.00	5.50
GO:0055114	oxidation-reduction process	659	73	0.0000	3.07	5.40
GO:0032259	methylation	270	26	0.0661	3.19	5.31
GO:0006567	threonine catabolic process	4	3	0.0013	3.33	5.33
GO:0052545	callose localization	10	3	0.0292	3.50	5.50
GO:0042274	ribosomal small subunit biogenesis	21	6	0.0407	3.50	5.67
GO:0007005	mitochondrion organization	59	9	0.0220	3.56	5.22
GO:0006749	glutathione metabolic process	13	3	0.0594	3.67	5.33
GO:0044205	'de novo' UMP biosynthetic process	5	5	0.0000	3.80	5.80
GO:0072488	ammonium transmembrane transport	12	8	0.0000	4.00	5.75
GO:0001731	formation of translation preinitiation complex	13	3	0.0594	4.00	5.67

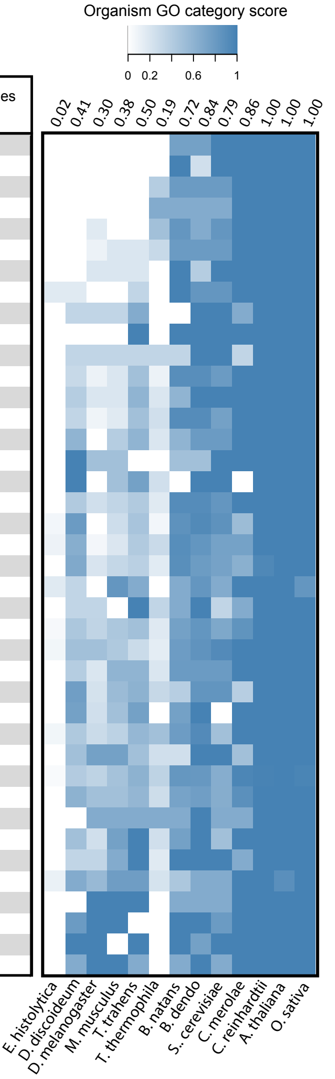

Figure S16. Cellular Component GO terms for *C. tetramitiformis* peptides enriched in non-phagocytes

GO.ID	Term-Cellular Compartment	# C.tet. peptides in GO category	# peptides in non-phagocyte enrichment	GO enrichment p-value	Phagocytes score	Non-phagocytes score
GO:0048046	apoplast	31	3	0.3657	2.00	5.33
GO:0009570	chloroplast stroma	157	38	0.0000	2.45	5.55
GO:0009507	chloroplast	506	79	0.0001	2.57	5.45
GO:0009295	nucleoid	13	3	0.0563	2.67	5.00
GO:0043231	intracellular membrane-bounded organelle	3500	277	0.1991	3.04	5.35
GO:0043227	membrane-bounded organelle	3606	282	0.2340	3.06	5.34
GO:0044425	membrane part	1643	112	0.6127	3.17	5.28
GO:0055044	symplast	83	6	0.5206	3.17	5.50
GO:0031224	intrinsic component of membrane	1426	106	0.2142	3.18	5.28
GO:0000775	chromosome, centromeric region	38	5	0.1200	3.20	5.20
GO:0005743	mitochondrial inner membrane	139	22	0.0002	3.25	5.33
GO:0005739	mitochondrion	498	59	0.0245	3.25	5.31
GO:0046930	pore complex	17	4	0.0264	3.25	5.25
GO:0005759	mitochondrial matrix	70	11	0.0082	3.33	5.58
GO:0032153	cell division site	19	3	0.1410	3.33	5.33
GO:0032040	small-subunit processome	16	5	0.0036	3.40	5.60
GO:0000325	plant-type vacuole	26	4	0.1019	3.60	5.60
GO:0034708	methyltransferase complex	17	3	0.1093	4.00	5.33

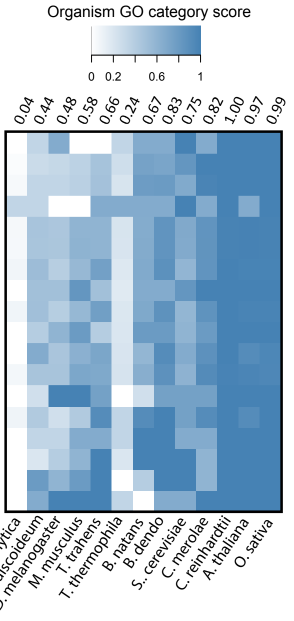

Figure S17. Molecular Function GO terms for *C. tetramitiformis* peptides enriched in non-phagocytes

GO.ID	Term-Molecular Function	# C.tet. peptides in GO category	# peptides in non-phagocyte enrichment	GO enrichment p-value	Phagocytes score	Non-phagocytes score
GO:0004197	cysteine-type endopeptidase activity	21	5	0.0145	0.29	5.00
GO:0016833	oxo-acid-lyase activity	7	3	0.0104	1.33	6.00
GO:0004072	aspartate kinase activity	3	3	0.0004	1.50	6.00
GO:0016836	hydro-lyase activity	26	6	0.0089	1.50	5.33
GO:0051539	4 iron, 4 sulfur cluster binding	44	6	0.0924	1.67	5.33
GO:0019202	amino acid kinase activity	4	4	0.0713	2.00	6.00
GO:0004175	endopeptidase activity	119	14	0.0449	2.28	5.17
GO:0016783	sulfurtransferase activity	10	3	0.0302	2.33	5.67
GO:0016829	lyase activity	135	20	0.1262	2.33	5.43
GO:0004609	phosphatidylserine decarboxylase activity	3	3	0.0004	2.50	5.00
GO:0016866	intramolecular transferase activity	30	9	0.0100	2.56	5.56
GO:0016830	carbon-carbon lyase activity	49	11	0.0148	2.58	5.50
GO:0035251	UDP-glucosyltransferase activity	12	5	0.0298	2.60	5.10
GO:0003885	D-arabinono-1,4-lactone oxidase activity	3	3	0.0004	2.67	5.00
GO:0009982	pseudouridine synthase activity	23	6	0.0047	2.67	5.33
GO:0016814	hydrolase activity, on C-N (not peptide) bonds	15	3	0.0878	2.67	5.67
GO:0048037	cofactor binding	238	27	0.0112	2.77	5.37
GO:0016853	isomerase activity	155	21	0.0464	2.90	5.48
GO:0020037	heme binding	55	10	0.0051	2.91	5.91
GO:0003838	sterol 24-C-methyltransferase activity	3	3	0.0004	3.00	6.00
GO:0015288	porin activity	5	3	0.0033	3.00	5.00
GO:0008526	phosphatidylinositol transporter activity	8	3	0.0157	3.00	5.33
GO:0015299	solute:proton antiporter activity	14	5	0.0492	3.00	5.43
GO:0016491	oxidoreductase activity	520	62	0.0363	3.04	5.39
GO:0016688	L-ascorbate peroxidase activity	7	5	0.0000	3.20	6.00
GO:0008762	UDP-N-acetylmuramate dehydrogenase activity	9	4	0.0025	3.20	5.00
GO:0004601	peroxidase activity	34	13	0.0067	3.21	5.93
GO:0004252	serine-type endopeptidase activity	34	6	0.0319	3.38	5.00
GO:0016796	exonuclease activity, RNA or DNA	25	4	0.1002	3.50	5.00
GO:0017057	6-phosphogluconolactonase activity	7	3	0.0104	3.67	5.67
GO:0005527	macrolide binding	20	5	0.0117	3.80	5.60
GO:0016884	carbon-nitrogen ligase activity, with GLN as N donor	12	5	0.0298	3.80	5.60
GO:0008519	ammonium transmembrane transporter activity	12	8	0.0000	4.00	5.75

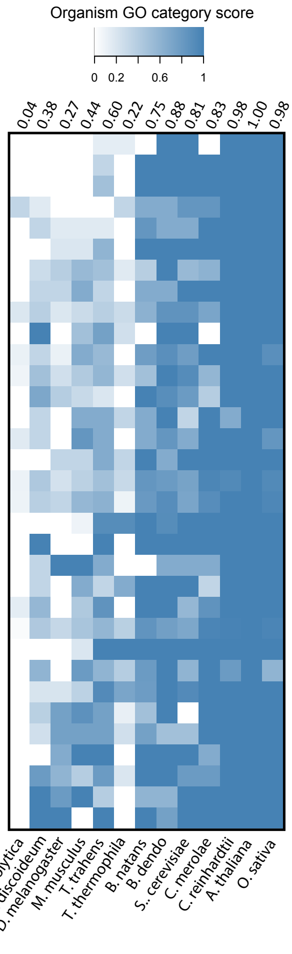

Supplement: Supplementary Data [file evv144_supplementary_data.zip › Cymbomonas_Suppl_Final_v2.pdf]
