# Supplementary material for: Comparative Genomics of a Bacterivorous Green Alga Reveals Evolutionary Causalities and Consequences of Phago-Mixotrophic Mode of Nutrition
Source: Genome Biol Evol. 2015 Jul 29;7(11):3047–61. doi: 10.1093/gbe/evv144 (PMC5741210; doi:10.1093/gbe/evv144)
Supplement: Supplementary Data [file supp_7_11_3047__index.html]

Comparative Genomics of a Bacterivorous Green Alga Reveals Evolutionary Causalities and Consequences of Phago-Mixotrophic Mode of Nutrition — Supplementary Data 

# Comparative Genomics of a Bacterivorous Green Alga Reveals Evolutionary Causalities and Consequences of Phago-Mixotrophic Mode of Nutrition

## Supplementary Data

files

- Supplementary Data - zip file
